# The Dual Role of Interleukin-6 in the Pathophysiology of Skeletal Muscle: Mechanisms, Challenges, and Therapeutic Prospects

**DOI:** 10.3390/ph19060868

**Published:** 2026-05-30

**Authors:** Yingyu Wang, Jitai Zhang, Jie Wang, Yijie Zhang, Jiacheng Sun, Jiahuan Gong, Xinlei Yao, Hualin Sun

**Affiliations:** 1 Jiangsu Key Laboratory of Tissue Engineering and Neuroregeneration, Key Laboratory of Neuroregeneration of Ministry of Education, Co-Innovation Center of Neuroregeneration, Medical School of Nantong University, Nantong University, Nantong 226001, China; yingyuwang1998@hotmail.com (Y.W.); 2424310006@stmail.ntu.edu.cn (J.Z.); 2022310019@stmail.ntu.edu.cn (J.W.); 2030110218@stmail.ntu.edu.cn (Y.Z.); 2331310083@stmail.ntu.edu.cn (J.S.); gongjh@ntu.edu.cn (J.G.); 2 Biological Physics Laboratory, Department of Physics and Astronomy, School of Natural Science, The University of Manchester, Manchester M13 9PL, UK

**Keywords:** interleukin-6, skeletal muscle, myokine, muscle atrophy, therapeutic strategy

## Abstract

Interleukin-6 (IL-6) is a cytokine with multiple biological effects. It plays a complex and seemingly paradoxical central role in both the physiological homeostasis and pathological processes of skeletal muscle. Under physiological conditions, particularly during acute exercise, IL-6 produced and secreted by the contracting skeletal muscle itself acts as an important “myokine.” It operates in an autocrine, paracrine, or endocrine manner to regulate systemic energy metabolism, insulin sensitivity, muscle regeneration, and adaptive hypertrophy. This function is crucial for the health benefits conferred by exercise. However, under various pathological conditions—such as cancer cachexia, sepsis, muscular dystrophy, denervation, disuse atrophy, and chronic inflammatory diseases—persistently elevated systemic or local IL-6 levels become a key mediator driving skeletal muscle atrophy, metabolic disorders, and functional decline. This review systematically elaborates on the dual role of IL-6 in skeletal muscle. It provides an in-depth analysis of its downstream signaling pathways (e.g., JAK/STAT, gp130, MAPK, PI3K-Akt) and upstream regulatory mechanisms (e.g., the Piezo1/KLF15 axis, calcium signaling, mitochondrial function, oxidative stress). A particular focus is placed on discussing the distinct biological effects of classical IL-6 signaling versus trans-signaling. Furthermore, we address current challenges in research and practice, including the cell specificity of IL-6 signaling, the complexity of its temporal regulation, the definition of physiological versus pathological concentrations, discrepancies between animal models and human diseases, and the plasticity of its function across different pathological contexts. Finally, this review explores the potential of targeting the IL-6 signaling pathway as a therapeutic strategy for skeletal muscle atrophy and related metabolic diseases. Potential interventions include IL-6/IL-6R monoclonal antibodies, JAK/STAT inhibitors, gp130 modulators, exercise interventions, and nutritional strategies. This aims to provide a theoretical foundation and novel perspectives for future translational research and clinical interventions.

## 1. Introduction

The evolving understanding of the biological role of interleukin-6 (IL-6) stands as a classic example in modern biomedicine of a journey from a partial to a comprehensive, and from a contradictory to a unified, perspective. For over three decades, IL-6 was firmly characterized as a key pro-inflammatory cytokine. This was due to its significant elevation in acute-phase responses, fever, and various autoimmune diseases and chronic inflammatory conditions [1]. However, this relatively narrow view was fundamentally challenged by a landmark discovery in the late 1990s. Researchers observed that plasma IL-6 concentration increases dramatically, by tens or even hundreds of times, in healthy individuals following high-intensity or prolonged exercise [2]. Critically, this rise occurs independently of the classic inflammatory cascade, as it is not accompanied by a significant increase in tumor necrosis factor-α (TNF-α) or interleukin-1β (IL-1β). Subsequent research confirmed that contracting skeletal muscle itself is the primary source of this exercise-induced surge in circulating IL-6. This discovery not only revealed the novel identity of skeletal muscle as an active endocrine organ but also formally established IL-6 as the first clearly identified “myokine” [3]. Myokines are proteins or peptides synthesized and secreted by muscle tissue, capable of exerting broad biological effects in an autocrine, paracrine, or endocrine manner.

**Figure 1:**
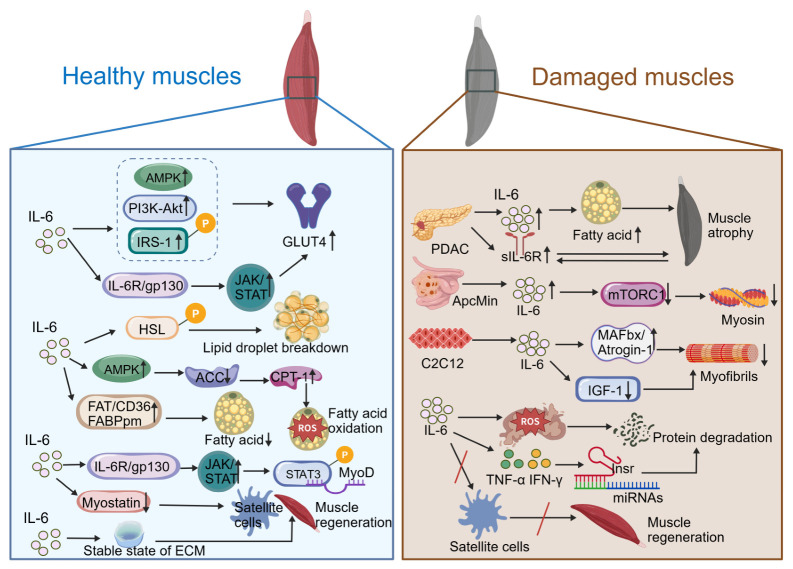
**The dual role of IL-6 in skeletal muscle homeostasis.** Under normal physiological conditions (left), IL-6 derived from the muscle acts as a muscle factor, enhancing glucose uptake, stimulating lipolysis, activating satellite cells and promoting tissue repair. In pathological conditions (right), the persistently elevated IL-6 drives muscle atrophy, mitochondrial dysfunction, and protein degradation. An upward arrow represents an upward expression, and a downward arrow represents a downward expression.

Since then, the paradigm of IL-6 in skeletal muscle biology has undergone a fundamental shift, entering a new era of dual-track progression—seemingly contradictory yet unified. On one hand, as an exercise-induced myokine, IL-6 demonstrates remarkable functions in metabolic regulation and homeostasis maintenance under physiological conditions (Figure 1). Specifically, acute elevation of myogenic IL-6 has been shown to promote glucose release and lipid mobilization from the liver and adipose tissue to meet energy demands during exercise. Concurrently, it enhances glucose uptake and insulin sensitivity in skeletal muscle by activating the AMP-activated protein kinase (AMPK) and phosphatidylinositol 3-kinase (PI3K)–Akt pathways. The underlying mechanisms involve promoting the translocation and expression of glucose transporter type 4 (GLUT4) [4,5]. For instance, studies indicate that exercise-induced elevation of IL-6 is essential for enhancing skeletal muscle insulin sensitivity and GLUT4 expression, whereas neutralizing IL-6 with antibodies abolishes these exercise benefits [5]. Additionally, myogenic IL-6 stimulates hepatic gluconeogenesis and regulates adipose tissue metabolism during the post-exercise recovery period, thereby contributing to the re-establishment of energy balance [6,7]. Beyond metabolic regulation, IL-6 also plays a constructive role in muscle repair and adaptation. Low-level or acute expression of IL-6 can promote the activation and proliferation of satellite cells via pathways such as signal transducer and activator of transcription 3 (STAT3), thereby supporting muscle regeneration [8]. Research further indicates that IL-6 interacts with insulin-like growth factor 1 (IGF-1) signaling to jointly regulate muscle growth and bone metabolism, with the mammalian target of rapamycin (mTOR) pathway likely serving as a key integration point [9]. Thus, under physiological conditions, IL-6 acts as an important myokine and constitutes a core regulatory network for metabolic adaptation and regenerative repair in skeletal muscle. However, this homeostasis-oriented physiological network is often subverted in pathological states, where IL-6 signaling instead drives inflammatory exhaustion and atrophy. This highlights the complex and highly context-dependent dual role of IL-6 in skeletal muscle pathophysiology.

On the other hand, under numerous chronic pathological conditions, persistently and abnormally elevated levels of IL-6—whether systemic or within the local microenvironment—are closely associated with progressive skeletal muscle wasting and dysfunction (Figure 1). This establishes IL-6 as a central molecular bridge linking chronic inflammation to muscle atrophy [10,11,12]. For example, in cachexia induced by malignancies such as pancreatic cancer, tumor cells not only secrete IL-6 themselves but also induce adipose tissue and muscle to form a positive feedback loop mediated by IL-6 trans-signaling. This loop jointly drives lipolysis, muscle fat infiltration, metabolic disturbances, and atrophy [13]. Similarly, in aging-related sarcopenia, elevated IL-6 levels accompanying the chronic low-grade inflammatory state (“inflamm-aging”) have been shown to inhibit peroxisome proliferator-activated receptor gamma coactivator 1-alpha (PGC-1α). This leads to excessive production of mitochondrial reactive oxygen species (ROS), thereby exacerbating protein degradation and muscle atrophy [12,14,15]. IL-6 is also recognized as a key mediator of muscle wasting in consumptive syndromes such as chronic kidney disease, heart failure, and chronic obstructive pulmonary disease [16]. Clinical observational studies provide strong supporting evidence: among healthy active women, serum IL-6 levels show a significant negative correlation with thigh muscle cross-sectional area, isometric strength, and bone mineral density at multiple sites. Notably, these associations remain independent of age, body mass index, and physical activity levels [17]. This double-edged, “friend-and-foe” dual identity makes IL-6 one of the most intriguing yet challenging molecules in the study of skeletal muscle pathophysiology.

The ultimate biological effects of IL-6 are determined by a complex and sophisticated regulatory network [18]. First, the context of its production is critical: acute, transient elevation (e.g., post-exercise) generally leads to beneficial metabolic adaptations and tissue repair, whereas chronic, sustained elevation (as seen in cancer and aging) often results in catabolism and tissue wasting. Second, the concentration matters: physiological levels (pM range) activate primarily classical signaling, while supraphysiological levels (nM range) can overwhelm regulatory mechanisms [19]. Third, the duration of exposure determines downstream effects: transient pulses promote adaptive responses, while persistent exposure leads to desensitization and pathological signaling [20]. Fourth, the signaling topology—classical versus trans-signaling—dictates which cell types respond and what transcriptional programs are activated [13]. The local microenvironment in which it acts—including coexisting cytokines, hormones, and surrounding cell types—profoundly influences IL-6 signaling output. Third, the specific signaling pathway activated is key to distinguishing its functions. IL-6 acts primarily through two modes: the “classical signaling” pathway, where IL-6 binds to membrane-bound IL-6 receptor (IL-6R) and glycoprotein 130 (gp130) on target cells, a pathway largely restricted to cells expressing membrane IL-6R, such as hepatocytes and certain leukocytes; and the “trans-signaling” pathway, where IL-6 complexes with soluble IL-6R (sIL-6R) and can activate almost all gp130-expressing cells (including skeletal muscle and adipocytes) [21]. Studies indicate that the latter is more closely associated with pro-inflammatory and pro-catabolic effects [13]. For example, in cancer cachexia, IL-6 trans-signaling serves as a central mechanism driving detrimental crosstalk between adipose and muscle tissues [13]. Finally, IL-6 signaling interacts with other signaling networks, such as synergy with TNF-α and IFN-γ, or antagonism with anabolic pathways like IGF-1/Akt/mTOR. These cross-talks further shape the eventual pathological or physiological phenotype [22]. Therefore, a deeper understanding of this multi-layered and dynamic regulatory network is essential for developing precise therapeutic strategies targeting IL-6 signaling imbalance in specific pathological conditions.

In recent years, research on the complex mechanisms of IL-6 in skeletal muscle has been increasingly deepened, and its finely regulated network is being progressively revealed [8,23,24]. Notably, the capacity of skeletal muscle to secrete IL-6 is myofiber type-dependent, with studies suggesting that slow-twitch muscle fibers may possess a greater potential for IL-6 secretion [25]. Upstream in the regulatory mechanism, alterations in mechanical signals represent a key control point. For instance, reduced intracellular calcium levels sensed by the Piezo1 channel can upregulate Krüppel-like factor 15 (KLF15), thereby inducing IL-6 expression and mediating immobilization-induced muscle atrophy. This provides a novel perspective on how changes in the mechanical environment influence muscle homeostasis via IL-6 [26]. Furthermore, epigenetic regulation is also involved. O-linked N-acetylglucosamine glycosylation (O-GlcNAcylation) can promote IL-6 transcription by modifying p65 in response to stress stimuli such as cold exposure [27]. Regarding interventional strategies, targeting the IL-6 signaling pathway has shown significant therapeutic potential. IL-6 receptor (IL-6R) monoclonal antibodies, such as tocilizumab, have been demonstrated to effectively improve muscle function or promote regeneration in experimental autoimmune myasthenia models and mouse models of Duchenne muscular dystrophy [28,29]. Meanwhile, small-molecule inhibitors, such as the JAK/STAT3 pathway inhibitor ruxolitinib, have also been confirmed to alleviate denervation- or cancer-induced muscle atrophy [8,30]. Beyond pharmacological approaches, non-pharmacological interventions—including exercise, nutritional supplements (e.g., magnesium), and natural compounds (e.g., cucurbitacin IIb)—have also been found to exert muscle-protective effects by modulating IL-6 production or signal transduction [31,32,33]. In summary, IL-6 plays a “double-edged sword” role in skeletal muscle homeostasis, with its specific effects highly dependent on the physiological or pathological context. Future research should focus on clarifying its complex regulatory network to develop precise intervention strategies for specific muscle disorders.

In summary, IL-6 plays a complex and multifaceted central role in skeletal muscle biology. The evolution of its perception—from a “pro-inflammatory factor” to a “pleiotropic myokine”—reflects not only the deepening of scientific understanding, but also highlights the core principle of context-dependent biology. A thorough dissection of its specific mechanisms of action, signaling pathway preferences, and regulatory networks under different physiological and pathological conditions is crucial. This knowledge is essential for developing novel and precise therapeutic strategies that target the IL-6 system to treat muscle wasting diseases, while preserving or even enhancing its beneficial metabolic functions. This review aims to systematically outline research progress in this field. Centered on the dual role of IL-6 in skeletal muscle, it explores the underlying molecular mechanisms, current challenges, and potential interventional strategies.

To ensure comprehensive and systematic coverage of the literature, we conducted a structured search of PubMed and Web of Science databases using the following keywords and combinations: ‘interleukin-6’ OR ‘IL-6’ AND ‘skeletal muscle’ OR ‘muscle atrophy’ OR ‘sarcopenia’ OR ‘muscle regeneration’. The search was limited to peer-reviewed articles published in English between 1988 and 2025, with priority given to original research articles, systematic reviews, and meta-analyses. Studies were included if they addressed molecular mechanisms of IL-6 signaling in skeletal muscle, the role of IL-6 in muscle physiology or pathology, or therapeutic interventions targeting the IL-6 pathway. Conference abstracts, unpublished data, and non-English articles were excluded. The broad scope of this review is intentional, as IL-6’s pleiotropic actions span multiple tissues (muscle, adipose, liver, immune system) and pathological contexts (cancer, aging, disuse, denervation, sepsis, metabolic disease). Rather than providing exhaustive coverage of every publication, this review synthesizes key mechanistic insights and highlights emerging therapeutic strategies, with a focus on studies that elucidate context-dependent signaling mechanisms and translational potential.

## 2. Physiological Roles of IL-6 in Skeletal Muscle

Exercise serves as a crucial physiological stimulus that induces the secretion of various myokines from skeletal muscle. Among these, IL-6 has attracted particular attention. Traditionally regarded as an inflammatory mediator, recent studies have revealed that exercise-induced IL-6 possesses distinct functions in metabolic regulation and tissue repair. It acts as a key signaling molecule in energy metabolism, coordinating energy distribution across multiple tissues during exercise. Meanwhile, it initiates regeneration programs following muscle injury. These findings not only redefine the physiological role of IL-6 but also offer a novel molecular perspective for understanding the health benefits of exercise.

### 2.1. Exercise-Induced IL-6 Secretion and Its Metabolic Regulation

**Figure 2:**
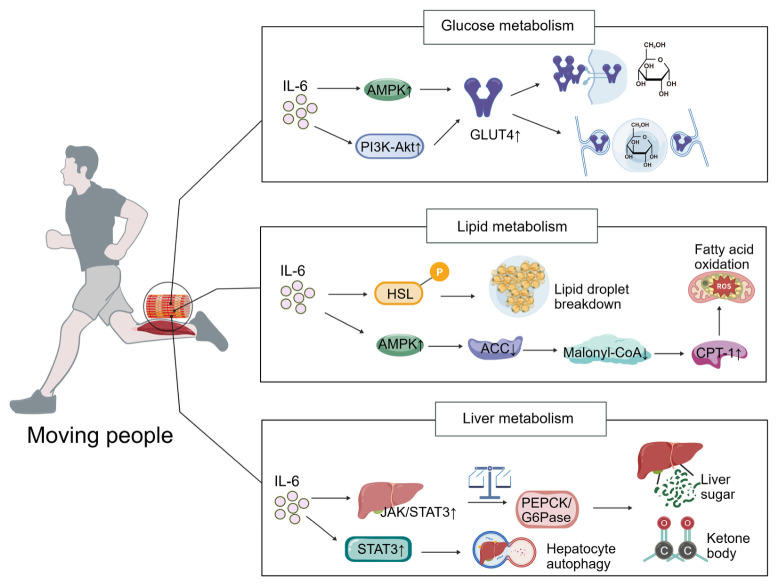
**Exercise-induced IL-6 secretion and its systemic metabolic regulation.** Contracting skeletal muscle releases IL-6 into the circulation, which acts on multiple tissues to coordinate energy homeostasis. In muscle, IL-6 enhances glucose uptake via AMPK and PI3K-Akt pathways and stimulates lipolysis via AMPK-ACC-CPT-1 axis. In the liver, IL-6 activates JAK/STAT3 signaling to promote gluconeogenesis and ketogenesis. An upward arrow represents an upward expression, and a downward arrow represents a downward expression.

Skeletal muscle contraction represents one of the most potent physiological stimuli for inducing IL-6 production from the myofibers themselves (muscle-derived IL-6, or ‘myokine’). Its secretion level is closely related to exercise intensity, duration, and muscle glycogen availability. When muscle glycogen content is low, IL-6 gene transcription and protein release are markedly enhanced. This suggests that IL-6 may act as an “energy sensor,” orchestrating a shift toward alternative fuel sources when carbohydrate availability is insufficient [2]. Recent studies further reveal that IL-6 not only serves as a key mediator in metabolic regulation but also plays a central role in multi-tissue energy homeostasis (Figure 2).

#### 2.1.1. Promotion of Glucose Metabolism

Exercise-induced IL-6 acts as a critical myokine, playing a central role in regulating glucose metabolism in skeletal muscle. IL-6 can significantly enhance glucose uptake in skeletal muscle. The mechanism primarily involves activating the AMP-activated protein kinase (AMPK) signaling pathway. AMPK functions as a cellular energy sensor and is activated during exercise. This activation promotes the translocation of glucose transporter type 4 (GLUT4) to the cell membrane, thereby increasing cellular glucose permeability [4,34]. Studies show that following acute IL-6 infusion or exercise, IL-6 can, in an autocrine manner, promote the expression of its own receptor (IL-6R) and downstream signaling molecules. This creates a positive feedback loop that amplifies its stimulatory effect on glucose uptake [5,35]. Recent research further reveals that IL-6 can also enhance insulin signaling by activating the phosphatidylinositol 3-kinase-protein kinase B (PI3K-Akt) pathway. This process not only improves insulin sensitivity but also increases the efficiency of glucose transmembrane transport by promoting the peripheral migration of GLUT4-containing vesicles [4]. Furthermore, IL-6 reinforces the insulin signaling pathway by inducing the phosphorylation of insulin receptor substrate 1 (IRS-1). This contributes to improved systemic glucose homeostasis after exercise [36]. It is noteworthy that IL-6’s regulation of glucose metabolism exhibits tissue specificity. Its promoting effect in skeletal muscle stands in stark contrast to its effects in the liver, highlighting its characteristic role in multi-organ coordination.

At the molecular level, IL-6 activates the downstream JAK/STAT signaling pathway through its receptor complex (IL-6R/gp130), particularly promoting the phosphorylation of STAT3, which regulates the expression of genes related to glucose metabolism [37]. Simultaneously, IL-6 can modulate key enzymes involved in glucose metabolism, such as pyruvate dehydrogenase (PDH), by influencing intracellular calcium concentrations, thereby finely tuning the process of glucose oxidation [38]. These findings reveal that IL-6 plays multiple roles in exercise-induced metabolic improvements, acting not only as an energy sensor but also as a core metabolic signaling molecule. However, under pathological conditions, persistent or abnormal activation of IL-6 signaling may lead to markedly different outcomes. For instance, in disuse-induced muscle atrophy, activation of the Piezo1/KLF15/IL-6 axis mediates muscle wasting triggered by decreased cytoplasmic calcium levels [26]. In cancer cachexia, tumor-derived IL-6 forms a feed-forward loop through trans-signaling across tissues (tumor, adipose, muscle), driving lipolysis and muscle atrophy [13]. Clinical observations have also shown that serum IL-6 levels are negatively correlated with skeletal muscle cross-sectional area and muscle strength in women [17]. Moreover, in models of muscle atrophy induced by denervation [8] or sepsis [14], inhibiting the IL-6/JAK/STAT3 pathway effectively attenuates muscle loss. Together, this evidence demonstrates that the role of IL-6 in skeletal muscle exhibits a distinct “double-edged sword” character: acute, moderate secretion (e.g., after exercise) serves as a key mediator of metabolic adaptation, whereas chronic, excessive production becomes a central driver of muscle wasting and pathological remodeling. This dual role, along with its complex regulatory networks—including classical signaling, trans-signaling, and crosstalk with other cytokines/pathways—constitutes the main challenges and opportunities in developing IL-6-targeted therapeutic strategies for skeletal muscle disorders. Therefore, a deeper dissection of the precise regulatory mechanisms of IL-6 signaling in specific physiological and pathological contexts is a crucial prerequisite for realizing its targeted therapeutic potential.

#### 2.1.2. Stimulation of Lipolysis

Exercise-induced IL-6 plays a pivotal role in lipid mobilization. Studies indicate that IL-6 significantly enhances the whole-body fat oxidation rate. This effect is primarily achieved by stimulating lipolysis within skeletal muscle itself, rather than through direct action on adipose tissue [39]. Specifically, during exercise, IL-6 promotes the hydrolysis of intramuscular triglycerides. This increases the availability and oxidation of fatty acids within the muscle, thereby providing energy for prolonged physical activity. Consequently, IL-6 can be viewed as a crucial signaling molecule linking exercise to energy supply. At the molecular level, IL-6 activates the phosphorylation of hormone-sensitive lipase (HSL), which promotes the breakdown of intracellular lipid droplets [40]. Concurrently, via the AMPK-mediated signaling pathway, IL-6 inhibits the activity of acetyl-CoA carboxylase (ACC). This reduces malonyl-CoA levels, thereby relieving the inhibition of carnitine palmitoyltransferase 1 (CPT-1). The subsequent promotion of fatty acid entry into mitochondria facilitates β-oxidation [14]. This coordinated series of responses ensures the efficient utilization of fatty acids and the smooth progression of energy metabolism during exercise.

It is noteworthy that IL-6’s regulation of fat metabolism exhibits tissue-specific differences. In skeletal muscle, IL-6 upregulates the expression of fatty acid transport proteins (e.g., FAT/CD36, FABPpm), thereby increasing fatty acid uptake and utilization [41]. In adipose tissue, the role of IL-6 is more complex. It may indirectly influence systemic lipid metabolism by modulating the secretion of adipokines such as adiponectin [42]. Furthermore, recent research has found that IL-6 can also affect mitochondrial biogenesis and fatty acid oxidation capacity by regulating the expression of peroxisome proliferator-activated receptor gamma coactivator 1-alpha (PGC-1α). This mechanism is particularly important in exercise-induced metabolic adaptation [14]. These findings reveal that IL-6 has multi-layered and multi-tissue networked functions in metabolic regulation. During exercise, IL-6-mediated lipolysis is tightly coupled with energy demands. When intramuscular glycogen stores are low, IL-6 secretion increases. By promoting fat mobilization, it provides an alternative energy source for the muscles. This mechanism is particularly significant in endurance exercise [43]. Additionally, IL-6 can influence post-exercise energy balance by modulating appetite-related hormones such as ghrelin, reflecting its multifaceted role in energy homeostasis. In summary, under exercise stress, IL-6 serves dual roles as both an energy sensor and a metabolic modulator. Its precise regulation is crucial for maintaining metabolic health.

#### 2.1.3. Regulation of Hepatic Metabolism

As an endocrine factor released into the bloodstream by exercising skeletal muscle, IL-6 acts on the distal liver to precisely regulate hepatic glucose output and systemic energy homeostasis. Studies have shown that skeletal muscle-specific IL-6 knockout (IL-6 MKO) mice exhibit significant abnormalities in hepatic glucose metabolism regulation after exercise. This finding directly confirms the indispensable role of muscle-derived IL-6 in exercise-mediated liver-muscle crosstalk [43,44]. At the molecular mechanism level, IL-6 primarily activates the JAK/STAT3 signaling pathway within hepatocytes. This activation subsequently regulates the expression of key glucose metabolism enzymes, such as phosphoenolpyruvate carboxykinase (PEPCK) and glucose-6-phosphatase (G6Pase). Notably, this effect is finely and specifically modulated during exercise, which is fundamentally distinct from the regulatory mechanism observed during fasting [44]. Furthermore, IL-6 can regulate hepatic ketone body metabolism by promoting the production of β-hydroxybutyrate in hepatocytes during prolonged exercise. This process provides alternative energy substrates for peripheral tissues and involves IL-6-mediated optimization of mitochondrial fatty acid oxidation capacity and energy metabolism efficiency in hepatocytes [45]. Recent research has also found that the IL-6/STAT3 signaling pathway can activate autophagy in hepatocytes during exercise. This promotes the mobilization and reuse of intracellular energy reserves, a mechanism that shows significant potential for improving metabolic diseases such as non-alcoholic fatty liver disease [46]. Moreover, IL-6 is widely involved in regulating the crosstalk between the liver and adipose tissue. For instance, it indirectly promotes the browning of white adipose tissue and energy expenditure by influencing hepatokines like fibroblast growth factor 21 (FGF21). Consequently, IL-6 plays a key role in the exercise-induced improvement of systemic metabolism [47]. These discoveries not only deepen our understanding of the metabolic benefits of exercise but also provide novel insights and therapeutic targets for interventions against metabolic diseases.

### 2.2. Positive Effects on Muscle Regeneration and Repair

Beyond acute metabolic regulation, IL-6 exerts a constructive role in the repair and regeneration of muscle tissue following injury. After muscle damage, satellite cells located in the basal lamina of muscle fibers become activated. They then proliferate and differentiate into new myocytes to repair the injury. Recent studies have further elucidated the central regulatory role of exercise-induced IL-6 in this process. This is particularly evident in activating satellite cells and coordinating tissue remodeling.

#### 2.2.1. Activation of Satellite Cells

The regenerative capacity of skeletal muscle primarily relies on the activation and proliferation of its key stem cell population, the satellite cells. This process is critically regulated by exercise-induced IL-6 [48]. Exercise, particularly eccentric contractions or high-intensity interval training, rapidly induces the local production of IL-6 within skeletal muscle. Notably, this IL-6 is not merely a byproduct of inflammation. Instead, it functions as an important myokine, acting in an autocrine or paracrine manner on satellite cells to initiate the repair program. The core mechanism involves IL-6 binding to its receptor complex (IL-6R/gp130), which activates the downstream JAK/STAT3 signaling pathway. Activated STAT3 is phosphorylated and dimerizes, then translocates into the nucleus. There, it directly binds to the promoter regions of myogenic regulatory factors, such as MyoD, thereby initiating the myogenic differentiation program [49,50]. Furthermore, sustained STAT3 activation is crucial for maintaining the proliferative state of satellite cells post-injury, preventing their premature differentiation. This ensures an adequate cell pool for subsequent regeneration. Further studies indicate that exercise-induced IL-6 production is inversely correlated with initial intramuscular glycogen levels. This suggests that energy stress may be an upstream trigger, thereby closely linking the metabolic state of muscle to its regenerative potential [6,19]. Beyond the classic JAK/STAT3 pathway, IL-6 can also create a favorable microenvironment for satellite cell activation and muscle regeneration by suppressing the expression of myostatin, a negative regulator of muscle growth [37]. Finally, studies in interleukin-6 knockout (IL-6 KO) mouse models provide direct evidence. These mice exhibit delayed muscle regeneration after injury, with attenuated satellite cell activation and proliferative responses. This is accompanied by impaired expression of insulin-like growth factor 1 (IGF-1) and activation of the downstream Akt/mTOR signaling pathway. These findings fully demonstrate that endogenous IL-6 is indispensable for initiating an effective skeletal muscle regeneration program [49]. In summary, exercise-induced IL-6 precisely regulates satellite cell function through multiple signaling pathways. It constitutes a central molecular hub connecting muscle activity, metabolism, and regenerative capacity.

#### 2.2.2. Orchestrating Tissue Remodeling

Successful skeletal muscle regeneration relies not only on the formation of new myofibers but also on the precise and coordinated remodeling of the extracellular matrix (ECM). The ECM provides structural support for regenerating myofibers and serves as a crucial signaling platform regulating cellular behavior. In this process, IL-6 acts as a “coordinator” in maintaining ECM homeostasis. Studies show that in models of muscle overload-induced hypertrophy, IL-6-deficient mice exhibit abnormal collagen deposition and interstitial fibrosis, accompanied by upregulated expression of transforming growth factor-β (TGF-β). This suggests that the absence of IL-6 leads to an imbalance between ECM synthesis and degradation, thereby impairing the regenerative microenvironment [51]. Conversely, exercise-induced IL-6 production prevents such aberrant fibrosis through multiple mechanisms. On the one hand, IL-6 can modulate the expression of matrix metalloproteinases (MMPs) and their tissue inhibitors (TIMPs), promoting appropriate ECM degradation and renewal. This creates space for the integration of new myofibers and angiogenesis [52]. On the other hand, IL-6 can influence the polarization of immune cells, such as macrophages, steering them toward an M2 phenotype with anti-inflammatory and reparative functions. Consequently, it helps shape an immune microenvironment conducive to tissue repair rather than chronic fibrosis [52]. Furthermore, IL-6 can inhibit the excessive activation of fibroblasts, reducing their abnormal collagen production. Human studies have also confirmed that elevated local IL-6 levels in muscle post-exercise are closely associated with subsequent superior structural and functional recovery [43]. Notably, the IL-6 trans-signaling pathway may play a unique and finely tuned regulatory role in coordinating communication among myofibers, fibroblasts, and immune cells to accomplish the complex task of tissue remodeling [53,54]. In summary, through its multidimensional and multicellular fine-tuning, IL-6 plays an indispensable role in maintaining ECM homeostasis and promoting functional muscle regeneration.

### 2.3. Muscle Type-Dependent Secretion Differences

The secretory capacity of IL-6 varies significantly among different types of skeletal muscle fibers. This variation is primarily determined by the metabolic characteristics, contraction patterns, and intrinsic molecular regulatory networks of the muscle fibers. Studies show that under ex vivo culture conditions, the soleus muscle, predominantly composed of slow-twitch oxidative fibers, can be effectively induced to release IL-6. In contrast, the extensor digitorum longus muscle, which consists mainly of fast-twitch glycolytic fibers, lacks this ability [25]. This secretory heterogeneity is closely related to the metabolic profiles of the fibers. Oxidative fibers are rich in mitochondria and rely primarily on fatty acid oxidation for energy. Their higher metabolic flexibility and calcium ion regulatory capacity may facilitate the activation of IL-6 synthesis and secretion pathways. For instance, during muscle contraction, the frequency and amplitude of calcium transients in slow-twitch fibers may differentially initiate IL-6 transcription and release, potentially through signaling pathways such as Ca^2+^/calmodulin-dependent kinases [14]. Therefore, muscle fiber type serves not only as a basis for structural and functional classification but also profoundly influences the secretory behavior of inflammation-related factors.

At the molecular level, beyond calcium signaling pathways, the fiber type-specific secretion of IL-6 is regulated by key transcription factors and epigenetic mechanisms. Peroxisome proliferator-activated receptor gamma coactivator 1-alpha (PGC-1α), which is highly expressed in oxidative muscle fibers, not only regulates mitochondrial biogenesis but also indirectly modulates IL-6 expression by influencing the activity of inflammatory transcription factors such as nuclear factor kappa B (NF-κB) and activator protein 1 (AP-1) [54]. Moreover, the phosphorylation level of signal transducer and activator of transcription 3 (STAT3) may differ among muscle fiber types, thereby affecting the autocrine and paracrine feedback loops of IL-6. Recent studies have also shown that microRNAs (e.g., miR-497-5p) are downregulated during IL-6-induced myotube atrophy. Their differential distribution in slow- and fast-twitch muscles may further modulate IL-6 signaling output [54]. These multi-layered and intertwined regulatory mechanisms collectively determine the expression patterns of IL-6 across different muscle fiber types.

Receptor distribution is another important factor contributing to secretory differences. Oxidative muscle fibers may exhibit enriched surface expression of IL-6 receptor complexes (e.g., gp130), rendering them more sensitive to local IL-6 signals and establishing positive feedback regulation. Meanwhile, other members of the myokine network, such as irisin and brain-derived neurotrophic factor (BDNF), also show fiber type-dependent expression. They may cooperate with IL-6 to coordinate metabolic adaptation and immune responses in muscle [47]. Notably, pathological conditions or external stimuli (e.g., exercise, exposure to inflammatory cytokines) can further amplify such secretory differences. For instance, in idiopathic inflammatory myopathies, aberrant IL-6 production is associated with intramuscular glycosylation deficiencies, and its expression pattern closely correlates with disease prognosis [55]. Therefore, a deeper understanding of the regulatory mechanisms governing IL-6 secretion in different muscle fiber types will not only help clarify its precise role in muscle physiology and pathology but also provide a theoretical foundation for developing fiber-type-specific interventions.

### 2.4. Synthesis and Commentary

The evidence presented in this section firmly establishes exercise-induced, muscle-derived IL-6 as a bona fide myokine with pleiotropic metabolic and regenerative functions. Several key insights emerge from the collective data. First, the magnitude of IL-6 release during exercise is inversely correlated with muscle glycogen content [36,56], positioning IL-6 as an energy-sensing signal that mobilizes alternative fuel sources when carbohydrate availability is limited. Second, the beneficial effects of exercise-induced IL-6 on glucose metabolism appear to be AMPK-dependent and PI3K-Akt-mediated, distinguishing this pathway from the JAK/STAT3-dominant effects seen in pathological states [4]. Third, the regenerative functions of IL-6—particularly satellite cell activation—require careful temporal regulation; both deficiency and chronic elevation impair regeneration [54,57].

However, important questions remain unresolved. Does muscle fiber type-specific IL-6 secretory capacity [25] translate to differential metabolic or regenerative responses in humans? Are the beneficial effects of exercise-induced IL-6 mediated entirely by classical signaling, or does trans-signaling contribute under certain conditions? The development of muscle-specific IL-6 knockout mice has been invaluable [7], but these models do not distinguish between IL-6’s autocrine effects on myofibers versus paracrine effects on adjacent satellite cells or immune cells. Future studies using conditional, cell-type-specific knockout systems will be essential to dissect these mechanisms.

## 3. The Role of IL-6 in Pathological Processes of Skeletal Muscle

**Figure 3:**
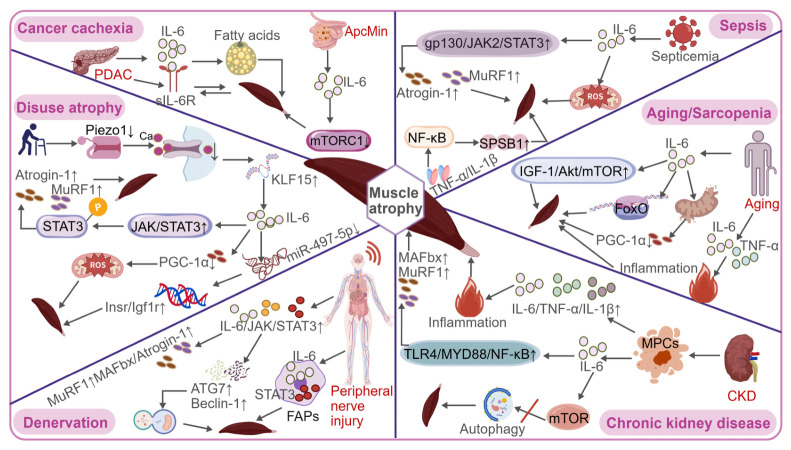
**IL-6 as a central mediator of muscle atrophy across diverse pathologies.** IL-6 contributes to muscle wasting through context-specific mechanisms in cancer cachexia (via trans-signaling and tissue crosstalk), disuse atrophy (via Piezo1/KLF15 axis), denervation (via sustained STAT3 activation), sepsis (via mitochondrial ROS), aging (via inflamm-aging), and chronic kidney disease (via uremic toxins and inflammation). An upward arrow represents an upward expression, and a downward arrow represents a downward expression.

Skeletal muscle atrophy is a common feature that severely impacts patient quality of life and prognosis across multiple pathological states. From cancer cachexia to chronic kidney disease, and from disuse atrophy to inherited muscular disorders, IL-6 has been identified as a core driver in these seemingly distinct pathological processes. As a pleiotropic cytokine, IL-6 profoundly influences skeletal muscle mass and functional homeostasis through multiple mechanisms (Figure 3). These include mediating tissue crosstalk, regulating the balance of protein synthesis and degradation, and affecting mitochondrial function. Elucidating the mechanisms of IL-6 in different muscle atrophy contexts is crucial for developing broad and effective intervention strategies.

### 3.1. Pivotal Role in Cancer Cachexia

Cancer cachexia is a syndrome characterized by the progressive wasting of skeletal muscle and adipose tissue, severely impacting patient quality of life and survival. In this complex pathological process, tumor-derived IL-6 and IL-6 from tumor-infiltrating immune cells act as core drivers. Through various molecular mechanisms, it directly or indirectly promotes skeletal muscle atrophy and metabolic dysregulation, constituting a key link in the initiation and progression of cachexia.

#### 3.1.1. Inter-Tissue Crosstalk and the Vicious Cycle

Research in malignancy models, such as pancreatic ductal adenocarcinoma (PDAC), has elucidated that tumor cells can initiate an inter-tissue feedforward loop mediated by IL-6 trans-signaling. The core of this cycle lies in the fact that tumor-derived IL-6 acts not only locally but also systemically. It triggers destructive crosstalk between adipose tissue and skeletal muscle, thereby driving systemic pathological changes in metabolism and structure [13]. Specifically, elevated IL-6 levels in the tumor microenvironment activate adipocytes, inducing lipolysis and the release of large amounts of free fatty acids (e.g., palmitate) into the circulation. Concurrently, the concentrations of IL-6 and its soluble receptor (sIL-6R) are significantly increased in the tumor itself, affected muscle, and peripheral blood. This creates conditions for the widespread activation of the trans-signaling pathway [13]. Thus, IL-6 plays a key initiating role in reshaping the systemic lipid metabolism network. These free fatty acids released from adipose tissue, in concert with circulating IL-6, act on skeletal muscle cells. This leads to abnormal intracellular lipid deposition (i.e., myosteatosis), disruption of metabolic homeostasis, and ultimately results in muscle fiber atrophy and functional loss [13]. Consequently, skeletal muscle is not merely a target of metabolic disorder but also a key site manifesting energy imbalance and structural damage. Importantly, the compromised skeletal muscle is not passively affected. It itself releases sIL-6R, which in turn feeds back to and enhances lipolysis in adipocytes. This forms a self-amplifying and continually worsening destructive cycle [13]. This positive feedback mechanism not only explains, from a kinetic perspective, why tissue wasting persists and progresses in cachexia but also highlights the central role of IL-6 signaling in coordinating pathological communication across multiple organs. Recent genetic evidence further supports the critical nature of this pathway. In PDAC patient-derived xenograft models, the absence of IL-6 significantly attenuated adipose tissue wasting and completely prevented the phenotypes of muscle steatosis, metabolic dysregulation, and atrophy [13]. This provides direct theoretical and experimental rationale for therapeutic strategies targeting the IL-6 signaling axis.

#### 3.1.2. Inhibition of Protein Synthesis

Beyond driving a vicious cycle between tissues, chronically elevated IL-6 levels can also directly target the anabolic processes in skeletal muscle to inhibit protein synthesis. In the ApcMin/+ colorectal cancer mouse model, systemic IL-6 overexpression significantly suppressed the activity of the mammalian target of rapamycin complex 1 (mTORC1) in a dose-dependent manner [58]. mTORC1 is a central signaling hub regulating cell growth and protein synthesis; its inhibition directly leads to reduced synthesis of key structural proteins, such as myosin heavy chain. Notably, this process depends on the activation of AMP-activated protein kinase (AMPK) and is independent of signal transducer and activator of transcription 3 (STAT3) signaling. This reveals a novel mechanism whereby IL-6 interferes with anabolism through an energy-sensing pathway (AMPK) [58]. Therefore, IL-6 may directly impact the balance of muscle protein metabolism by regulating cellular energy status.

More in-depth studies indicate that the inhibitory effect of IL-6 on protein synthesis emerges early in the development of cachexia. Even during the pre-tumor stage, systemic IL-6 overexpression significantly suppressed protein synthesis induced by eccentric contraction exercise [20]. This suggests that, prior to the onset of overt weight loss and muscle wasting, IL-6 already impairs the ability of skeletal muscle to respond to normal anabolic stimuli, such as mechanical loading. This provides a potential early window for intervention in cachexia. Furthermore, this inhibitory effect is independent of the activation status of mTORC1, hinting at the involvement of other, not yet fully elucidated, downstream effectors in this process [20]. These findings underscore that blocking IL-6 signaling before clinical symptoms appear may help preserve muscle metabolic function.

Recent research has further expanded our understanding of the mechanisms underlying IL-6-mediated inhibition of anabolism. In the C2C12 myotube model, IL-6 upregulates the expression of E3 ubiquitin ligases (e.g., MAFbx/Atrogin-1) and enhances the activity of the ubiquitin-proteasome system, thereby accelerating the degradation of myofibrillar proteins [27]. Additionally, IL-6 can downregulate the insulin-like growth factor 1 (IGF-1) signaling pathway, further weakening the drive for anabolism [9]. Mitochondrial dysfunction is also involved. IL-6 induces the production of mitochondrial reactive oxygen species (ROS), leading to oxidative stress, which subsequently activates proteolytic pathways and inhibits protein synthesis [14]. In the context of cancer cachexia, IL-6 acts synergistically with other factors in the tumor microenvironment (e.g., TNF-α, IFN-γ). This synergy can induce alterations in the expression of muscle-specific microRNAs (e.g., miR-497-5p). These microRNAs further exacerbate the imbalance between protein synthesis and degradation by regulating target genes such as the insulin receptor (Insr) [54]. Recent evidence also shows that IL-6 can impair muscle regenerative potential by affecting satellite cell function, thereby compromising long-term muscle mass maintenance [37]. In summary, IL-6 promotes the pathological progression of skeletal muscle atrophy through multi-level, multi-pathway synergistic actions.

### 3.2. Novel Mechanisms in Disuse-Induced Muscle Atrophy

In the development of disuse-induced muscle atrophy, the upregulation of IL-6 expression is a key pathological event. Its regulatory mechanism has recently been elucidated through a novel signaling axis—the Piezo1/KLF15/IL-6 axis. Under muscle disuse conditions, such as limb immobilization or weightlessness, the expression of the mechanosensitive ion channel Piezo1 decreases. This leads to a sustained reduction in intracellular calcium concentration below baseline levels. This chronic “low-calcium signal” state acts as a trigger, upregulating the expression of the transcription factor KLF15, which in turn directly promotes IL-6 transcription and synthesis. Genetic knockout or pharmacological blockade experiments demonstrate that muscle-specific deletion of KLF15 or systemic deletion of IL-6 effectively protects mice from disuse-induced atrophy. This signaling axis has also been validated in human samples, establishing the “low-calcium trigger event” as a new paradigm in muscle atrophy [26]. This discovery provides a crucial perspective for understanding the initial signal transduction mechanism by which muscle disuse leads to atrophy.

The downstream role of IL-6 in disuse atrophy involves multiple cellular signaling pathways. Firstly, IL-6 binding to its receptor activates the JAK/STAT3 pathway, causing STAT3 phosphorylation and nuclear translocation. This subsequently upregulates the expression of the ubiquitin ligases Atrogin-1 and MuRF1, promoting the ubiquitination and degradation of key structural proteins, such as myosin heavy chains, thereby directly accelerating myofiber atrophy [8,59]. Furthermore, IL-6 induces mitochondrial dysfunction and increases the production of mitochondrial reactive oxygen species (ROS). Therefore, IL-6 coordinately drives muscle wasting through two major pathways: regulating protein degradation and impairing mitochondrial function.

Simultaneously, IL-6 can regulate the expression of specific microRNAs in skeletal muscle at the post-transcriptional level. For instance, it suppresses the expression of miR-497-5p, thereby enhancing the expression of genes related to the insulin-like growth factor pathway, such as Insr and Igf1r. However, in a prolonged high IL-6 environment, this feedback mechanism paradoxically leads to myotube atrophy, highlighting the complexity of IL-6 regulatory networks [54]. In in vivo models, systemic IL-6 overexpression can also directly inhibit muscle protein synthesis. Specifically, IL-6 reduces the basal protein synthesis rate and the synthesis response induced by eccentric contraction via the gp130 signaling pathway. This process involves the downregulation of mTORC1 signaling, further confirming the central role of IL-6 in negatively regulating muscle mass [20]. These studies suggest that interventions targeting different nodes downstream of IL-6 may provide multi-dimensional strategies for treating muscle atrophy.

### 3.3. Denervation-Induced Muscle Atrophy

Peripheral nerve injury leading to muscle denervation triggers rapid and severe muscle atrophy. The underlying molecular mechanisms involve a marked activation of inflammatory responses. Microarray analyses reveal that inflammatory-related cytokines are strongly upregulated in denervated skeletal muscle, with the IL-6/JAK/STAT3 signaling pathway exhibiting sustained activation [8]. Further studies indicate that denervation-activated STAT3-IL-6 signaling plays a central role in fibro-adipogenic progenitors (FAPs). Following denervation, FAPs abnormally accumulate in muscle and show persistent STAT3 activation. This leads to the secretion of high levels of IL-6, which promotes muscle fiber atrophy and fibrosis in a paracrine manner [59]. In both in vitro and in vivo models, IL-6 directly enhances myotube atrophy by activating the JAK/STAT3 pathway. The use of an IL-6 receptor antibody (such as tocilizumab), JAK1/2 inhibitors (such as ruxolitinib), or a STAT3 inhibitor (such as C188-9) effectively suppresses muscle atrophy. These treatments also reduce the expression of mitophagy-related genes, including PINK1, BNIP3, and LC3B [8]. Mechanistically, activation of the IL-6/JAK/STAT3 pathway upregulates the muscle-specific ubiquitin ligases MuRF1 and MAFbx/Atrogin-1, thereby accelerating protein degradation. Concurrently, this pathway induces mitophagy, reflected by increased expression of autophagy-related proteins such as ATG7 and Beclin-1. These changes ultimately lead to the loss of muscle fiber structure and function [8]. Moreover, transcriptomic analyses reveal that IL-6-induced muscle atrophy also involves immune receptor activation and energy metabolism dysfunction. Genes related to oxidative phosphorylation and the tricarboxylic acid cycle are downregulated. Transcription factors such as STAT3, NF-κB, and TP53 may act as downstream regulatory nodes that collectively drive the atrophic process [23]. It is noteworthy that aberrant IL-6 signaling following denervation is not confined to local muscle tissue. Similar mechanisms have been observed in models of spinal cord injury and amyotrophic lateral sclerosis, highlighting the broad role of this pathway in pathophysiology [59]. Thus, IL-6 promotes denervation-induced muscle atrophy through multiple layers of mechanisms, establishing it as a key molecule for understanding this pathological process.

### 3.4. Sepsis and Aging-Associated Muscle Atrophy

In sepsis models, IL-6 deficiency (IL-6 KO) significantly attenuates skeletal muscle atrophy and improves muscle fiber cross-sectional area and contractile function. This protective mechanism is associated with the upregulation of peroxisome proliferator-activated receptor gamma coactivator 1-alpha (PGC-1α) and the inhibition of mitochondrial reactive oxygen species (ROS) production [14]. Specifically, IL-6 activates the glycoprotein 130 (gp130)/JAK2/STAT3 signaling pathway. This activation induces the expression of muscle atrophy-related genes, such as Atrogin-1 and MuRF1, thereby promoting protein degradation [60]. Furthermore, during sepsis, IL-6 increases mitochondrial ROS production. This leads to oxidative stress and cellular damage, further exacerbating muscle loss [14]. Studies indicate that sepsis-induced muscle atrophy positively correlates with IL-6 levels. IL-6 affects muscle cell homeostasis through both autocrine and paracrine manners [60]. Notably, inflammatory cytokines, such as TNF-α and IL-1β, can upregulate the expression of SPSB1 in muscle cells via pathways like NF-κB. This subsequently inhibits TGF-β receptor II signaling, impairs myogenesis, and aggravates atrophy [61]. Together, these findings reveal the multifaceted role of IL-6 as a core inflammatory mediator in sepsis-associated muscle atrophy.

During the aging process, a state of chronic low-grade inflammation, termed “inflammaging,” accompanies elevated circulating IL-6 levels. This elevation is closely associated with decreased muscle strength and the development of sarcopenia [62,63]. With advancing age, persistently high IL-6 levels can disrupt muscle protein balance and contribute to muscle fiber loss. This occurs through the inhibition of the insulin-like growth factor 1 (IGF-1)/Akt/mTOR anabolic pathway and the concurrent activation of the FoxO transcription factor-dependent proteolytic pathway [64]. In patients with sarcopenia, serum IL-6 levels significantly correlate with reduced muscle mass and decreased grip strength. This suggests its role as a key biomarker of inflammaging [65]. Moreover, aging-associated increases in IL-6 can disrupt mitochondrial biogenesis and function, reducing PGC-1α expression. This further weakens muscle oxidative metabolic capacity and contractile performance [14]. Additionally, IL-6 impairs muscle cell function and promotes satellite cell senescence by inducing mitochondrial dysfunction and oxidative stress, thereby weakening muscle regenerative capacity [66]. Recent studies also found that immune cell infiltration and the local inflammatory microenvironment in aged muscle can sustain aberrant IL-6 secretion. This creates a positive feedback loop that continuously drives muscle atrophy [67]. Recent research further indicates that IL-6 acts synergistically with other inflammatory factors, such as TNF-α, to create a pro-inflammatory environment that further accelerates muscle loss [68]. In aged muscle, IL-6 not only acts directly on muscle fibers but also sustains a chronic inflammatory state by modulating immune cell infiltration and cytokine networks. This process continuously promotes muscle atrophy [64]. Therefore, IL-6 is not merely an indicator of aging-related muscle decline. It actively participates in its pathophysiological evolution through multiple molecular mechanisms.

### 3.5. Diabetic Muscle Atrophy

In diabetes mellitus (DM), skeletal muscle atrophy is a common complication. Its development and progression are closely associated with various cytokines, among which IL-6 plays a complex and critical role [69,70,71]. As a pleiotropic cytokine, IL-6 is secreted by contracting skeletal muscle under physiological conditions. It regulates muscle metabolism and insulin sensitivity in an autocrine/paracrine manner. However, in the diabetic state, its signaling pathways become aberrantly altered. This leads to an imbalance in the regulatory effects on skeletal muscle metabolism. Therefore, a thorough analysis of the functional switch of IL-6 between physiological and pathological conditions is essential for understanding the mechanisms of diabetic muscle atrophy.

Studies indicate that skeletal muscle cells in patients with type 2 diabetes exhibit reduced responsiveness to IL-6, manifesting as selective IL-6 resistance. Specifically, acute IL-6 exposure in myotubes from individuals with normal glucose tolerance enhances glycogen synthesis, glucose uptake, and promotes STAT3 phosphorylation. In contrast, in myotubes from type 2 diabetes patients, IL-6’s promoting effect on glucose metabolism is lost. Concurrently, STAT3 signaling pathway activation is impaired, accompanied by upregulated SOCS3 expression [72]. This selective resistance may be related to the chronic inflammation triggered by persistent hyperglycemia and a lipotoxic environment. Thus, impairment of the IL-6 signaling pathway under diabetic conditions is a key factor leading to the failure of metabolic regulation.

In the pathogenesis of diabetic muscle atrophy, IL-6 is involved in regulation through multiple mechanisms. First, chronic hyperglycemia and elevated free fatty acid levels can stimulate the sustained production of IL-6 by skeletal muscle, creating a low-grade inflammatory state. This subsequently activates the JNK and IKKβ/NF-κB signaling pathways, exacerbating insulin resistance [73]. Second, IL-6 can also disrupt muscle metabolism by affecting mitochondrial function. For example, it downregulates the expression of the mitochondrial fusion protein Mfn2. This leads to disorganization of the mitochondrial network and impaired oxidative phosphorylation, thereby promoting muscle protein degradation [74]. Together, these pathways indicate that IL-6 plays multiple pro-atrophic roles in diabetic muscle atrophy.

Notably, exercise-induced IL-6 release and pathologically sustained elevated IL-6 exhibit distinctly different biological effects. IL-6 transiently secreted by skeletal muscle during exercise exerts anti-inflammatory effects and improves glucose uptake via the AMPK signaling pathway. In the diabetic state, however, while basal IL-6 levels in skeletal muscle are chronically elevated, the capacity for its exercise-induced release may paradoxically be enhanced. This contradiction suggests a remodeling of tissue-specific IL-6 signal transduction under pathological conditions [75]. Therefore, distinguishing the source and release patterns of IL-6 is crucial for elucidating its role in muscle metabolism. Additionally, IL-6 influences the progression of diabetic muscle atrophy by regulating the muscle regeneration process. Research shows that IL-6 family cytokines can activate STAT3 signaling in satellite cells, promoting muscle regeneration. In the diabetic environment, however, persistently elevated IL-6 may lead to excessive activation of STAT3 signaling. This, in turn, can induce the expression of muscle atrophy-related genes, such as Atrogin-1 and MuRF-1 [76]. This further illustrates that the impact of IL-6 on muscle homeostasis is highly dependent on its expression level and the duration of signaling.

Recent studies continue to reveal new mechanisms and potential intervention strategies for IL-6 in diabetic muscle atrophy. For example, Salbutamol can ameliorate skeletal muscle atrophy in STZ-induced diabetic rats by reducing serum IL-6 levels [77]. Aspalathin improves insulin resistance and mitochondrial dysfunction by inhibiting the IL-6/TNF-α/PKC-θ inflammatory axis [78]. Furthermore, elevated IL-6 in a state of low-grade systemic inflammation is closely associated with reduced skeletal muscle mass in older adults [79]. In cellular models, a combined preparation of *K. pinnata* and metformin can modulate the expression of inflammatory factors including IL-6 [80]. High-intensity interval training (HIIT) can also ameliorate diabetic myopathy by modulating the myokine profile, including IL-6 [81]. These advances provide diversified perspectives for therapies targeting IL-6 signaling.

### 3.6. Muscle Atrophy in CKD

Skeletal muscle atrophy is a common and serious complication during the course of chronic kidney disease (CKD), closely associated with increased patient mortality. As a pleiotropic cytokine, interleukin-6 (IL-6) plays a critical dual role in this pathological process. Its mechanisms include promoting inflammatory responses, inducing oxidative stress, triggering mitochondrial dysfunction, and activating muscle protein degradation pathways. Therefore, a deeper understanding of the mechanisms by which IL-6 contributes to muscle atrophy in CKD is essential for developing targeted intervention strategies.

In the CKD setting, IL-6 originates from both local muscle tissue and systemic inflammation. Senescent muscle precursor cells (MPCs) are a key local source, secreting large amounts of pro-inflammatory factors such as IL-6 via the senescence-associated secretory phenotype (SASP). Studies show that in CKD mouse models, markers of MPC senescence, such as SA-β-gal and the DNA damage response marker γ-H2AX, are significantly increased. Concurrently, levels of SASP factors (e.g., IL-6, TNF-α, and IL-1β) are markedly elevated [82]. Furthermore, uremic serum can induce MPC senescence, thereby promoting IL-6 secretion [82]. Beyond local production, systemic inflammation also drives increased IL-6 levels. For example, elevated serum IL-6 in CKD patients is significantly correlated with decreased muscle strength and reduced exercise tolerance [83]. These findings suggest that targeting both local muscle and systemic IL-6 production may be a potential strategy to mitigate muscle atrophy.

At the molecular level, IL-6 primarily drives muscle atrophy by activating the TLR4/MYD88/NF-κB signaling pathway. This pathway upregulates the expression of muscle-specific atrophy-related proteins, MAFbx and MuRF1, thereby accelerating protein degradation [84]. In a CKD rat model, caffeic acid reduced IL-6 levels and effectively alleviated muscle atrophy by inhibiting the TLR4/MYD88/NF-κB pathway [84]. Meanwhile, IL-6 is also closely linked to oxidative stress and mitochondrial dysfunction. Uremic toxins, such as indoxyl sulfate (IS), can increase muscle IL-6 expression and upregulate myostatin and atrogin-1 through oxidative stress-mediated mechanisms, leading to reduced muscle mass [85]. In CKD mice, IS accumulation induces mitochondrial dysfunction alongside elevated IL-6. Mitochondria-targeted interventions, such as L-carnitine or teneligliptin, can ameliorate these abnormalities [86]. These findings indicate that IL-6 cooperatively promotes protein degradation and cellular energy imbalance through multiple signaling pathways.

Additionally, IL-6 exacerbates muscle loss by affecting vascular function and cellular autophagy. In CKD patients, endothelial dysfunction and peripheral arterial disease often coexist with sarcopenia and are accompanied by elevated serum IL-6 levels [87]. This suggests that IL-6 may worsen atrophy by impairing muscle microcirculation. On the other hand, in an inflammatory environment, IL-6 and other cytokines, such as TNF-α, can stabilize mTOR signaling and inhibit autophagy. The upregulation of calpain 6 further reinforces this effect [88]. Recent research also reveals that METTL3 promotes IL-6 expression by regulating the TLR4/NF-κB pathway through m6A RNA modification, thereby aggravating muscle oxidative stress and inflammation [89]. Thus, the role of IL-6 in muscle atrophy involves an extensive and interconnected network, spanning inflammation, metabolism, vascular function, and epigenetic regulation.

### 3.7. Hereditary Muscle Diseases

In the pathological progression of Duchenne Muscular Dystrophy (DMD), IL-6 serves as a key inflammatory factor. Its expression is significantly elevated in both muscle tissue and serum of patients and the mdx mouse model. This elevation constitutes a core feature of the chronic inflammatory microenvironment [90,91]. This sustained high expression of IL-6 stems not only from the local immune response triggered by muscle necrosis but is also closely linked to a systemic low-grade inflammatory state. Notably, IL-6 exhibits functional duality in hereditary muscle diseases. On one hand, it exacerbates inflammatory cell infiltration and muscle damage via the classical signaling pathway. On the other hand, under specific conditions, it may participate in regulatory immune responses through the trans-signaling pathway, highlighting the complexity of its role [29]. Therefore, a deep dissection of IL-6’s specific modes of action at different pathological stages is crucial for understanding the inflammatory regulatory network in DMD.

At the molecular mechanism level, IL-6 is primarily involved in DMD progression by activating the JAK/STAT3 signaling pathway. In the mdx mouse model, IL-6 binding to its receptor induces sustained phosphorylation of STAT3. This subsequently upregulates the expression of pro-fibrotic factors such as Transforming Growth Factor-β (TGF-β), promoting extracellular matrix deposition. Ultimately, this leads to the replacement of muscle fibers with fibrous connective tissue [92]. Furthermore, IL-6 can form a positive feedback loop with inflammatory factors like IL-1β. This synergistically enhances the activation of the NF-κB pathway, thereby amplifying the local inflammatory response. It also promotes the infiltration of neutrophils and M1 macrophages, exacerbating sarcolemmal instability and calcium influx [93]. The persistent activation of these signaling cascades forms an important basis for the progressive fibrosis and functional decline in DMD.

It is noteworthy that the role of IL-6 in muscle pathology shows significant stage dependency. In the early stages of the disease, IL-6 may exert beneficial effects by promoting satellite cell proliferation and muscle regeneration. However, as the disease progresses, its sustained high expression leads to exhaustion of regenerative potential and accelerates the fibrotic process [29]. This dynamic change is particularly evident in the more severely affected dystrophin/utrophin double-knockout (dKO) mouse model, where blocking the IL-6 receptor significantly promotes skeletal muscle regeneration and reduces fibrosis. Recent studies further reveal that IL-6 may participate in disease regulation by affecting mitochondrial function. In D2-mdx mice, inhibiting the mitochondrial fission protein Drp1 downregulates IL-6 expression. Concurrently, this improves muscle strength and reduces fibrosis, suggesting a potential link between IL-6 and mitochondrial dysfunction [94]. This provides a new perspective for intervening in IL-6-related pathology from the angle of energy metabolism.

Beyond its direct effects on muscle tissue, the impact of IL-6 on the neuromuscular system cannot be overlooked. In hippocampal neurons lacking dystrophin, IL-6 can lead to neuronal hyperexcitability by modulating GABAergic signaling. This may be one of the mechanisms underlying cognitive dysfunction in some DMD patients [95]. Another study shows that treating D2-mdx mice with the adiponectin receptor agonist ALY688 improves their recognition memory by reducing IL-6 levels in the hippocampus. This further confirms the important role of IL-6 in nerve-muscle crosstalk [96]. These findings expand the pathological implications of IL-6 in the multi-system involvement of DMD.

### 3.8. Synthesis and Commentary

The evidence reviewed in Section 3 unequivocally establishes IL 6 as a central mediator of skeletal muscle wasting across diverse pathological conditions–from cancer cachexia and denervation to sepsis, aging, diabetes, CKD, and hereditary muscle diseases. Several unifying themes emerge. First, chronically elevated IL 6 (whether systemic or local) is consistently associated with muscle atrophy, in stark contrast to the transient, beneficial IL 6 elevation after exercise. Second, the JAK/STAT3 pathway is a dominant downstream effector of pathological IL 6 signaling, driving expression of the E3 ubiquitin ligases Atrogin 1 and MuRF1 [8,97]. Third, trans signaling (IL 6/sIL 6R/gp130) appears to be the primary mode of action in cachexia and chronic inflammation, as opposed to the classical pathway that dominates in exercise [13].

However, several critical controversies remain. (1) Causality vs. association: In many human studies (e.g., sarcopenia, CKD), elevated IL 6 correlates with muscle loss, but it remains unclear whether IL 6 is a primary driver or an epiphenomenon of the underlying disease. (2) Source specificity: Most animal models do not distinguish between IL 6 produced by myofibers, immune cells, or tumor cells. The development of cell-type-specific IL 6-knockout mice is urgently needed. (3) Stage-dependent effects: In Duchenne muscular dystrophy, IL 6 may promote regeneration early but drive fibrosis later [29]. Such stage-dependent switching is poorly characterized in other conditions. (4) Human relevance: Many preclinical studies use supraphysiological IL 6 concentrations (ng/mL) that are rarely achieved in human disease [19]. Future research should prioritize human ex vivo systems (e.g., muscle organoids, biopsies from well-phenotyped patients) and use clinically relevant IL 6 doses.

## 4. Molecular Mechanisms of IL-6 Signal Transduction

IL-6 is a pleiotropic cytokine whose functional diversity largely stems from its complex and versatile signal transduction mechanisms. IL-6 initiates multiple downstream signaling pathways—including JAK/STAT, MAPK, and PI3K-Akt—by binding to receptor systems on the surface of target cells. Among these, classic signaling and trans-signaling represent the two most important forms through which IL-6 exerts its biological effects. Although both pathways share the common signal transducer gp130, differences in the receptor complexes engaged during initiation lead to distinct, and often opposing, biological outcomes [98]. A detailed understanding of the molecular intricacies of these two signaling modes is key to elucidating the divergent roles IL-6 plays in skeletal muscle physiology and pathology.

*Core versus Modulatory Pathways in IL-6 Signaling*.

To assist the reader in navigating the complex IL-6 signaling network, we propose the following hierarchical framework:

**Core (Major) Pathways**—Essential for IL-6’s primary biological effects in skeletal muscle: (1) **JAK/STAT3** (particularly STAT3): The canonical and most thoroughly characterized pathway. STAT3 phosphorylation, nuclear translocation, and transcriptional regulation of target genes (SOCS3, c-Myc, atrogenes) mediate both metabolic and atrophic effects. This pathway is considered the primary effector of IL-6 signaling [8,97]. (2) **gp130**: The shared signal-transducing receptor essential for both classical and trans-signaling. No IL-6 signal can transduce without gp130 dimerization [13]. (3) **Classical vs. Trans-signaling**: The mode of receptor engagement (mIL-6R vs. sIL-6R) determines cellular responsiveness and biological outcome. This distinction is fundamental to understanding IL-6’s dual roles [57].

**Modulatory (Peripheral) Pathways**—Fine-tune or amplify the core signal, but are not essential for IL-6’s primary actions: (1) **MAPK (ERK1/2, p38, JNK)**: These pathways modulate the intensity and duration of STAT3 signaling and contribute to specific outcomes such as cell proliferation or stress responses. They act as signal integrators rather than primary effectors [99,100]. (2) **PI3K-Akt**: Under specific conditions (e.g., exercise), IL-6 can activate this pathway to promote glucose uptake and insulin sensitivity. This effect is context-dependent and not universally observed across all IL-6 signaling scenarios [4]. (3) **NF-κB**: While NF-κB is a major regulator of IL-6 gene transcription (upstream), it is not a direct downstream effector of IL-6 signaling. This distinction is important for understanding the IL-6 regulatory network [101,102].

Throughout the following subsections, we highlight which pathways are core versus modulatory to help readers prioritize mechanistic understanding.

### 4.1. Classical Signaling and Trans-Signaling

**Figure 4:**
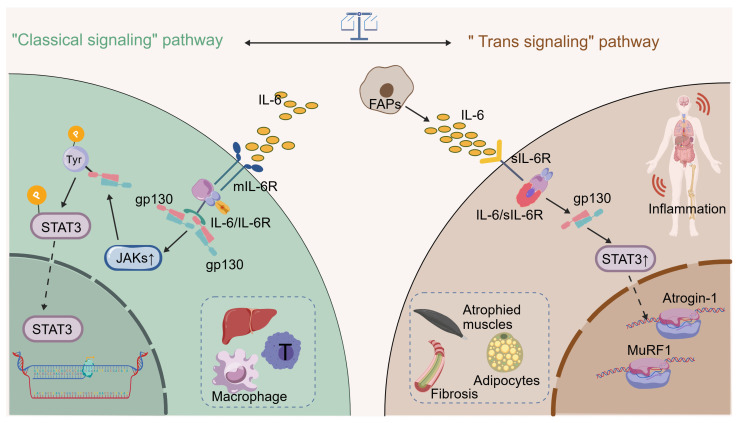
**Classical and trans-signaling pathways of IL-6.** In classical signaling (left), IL-6 binds to membrane-bound IL-6R (mIL-6R) and gp130 on target cells (e.g., hepatocytes, leukocytes). In trans-signaling (right), IL-6 forms a complex with soluble IL-6R (sIL-6R) that activates gp130 on cells lacking mIL-6R (e.g., myofibers, adipocytes). Trans-signaling is often associated with chronic inflammatory and catabolic states.

Within the signal transduction mechanisms of IL-6, the classical signaling pathway represents its initially elucidated mode of action. This pathway proceeds through an ordered sequence of steps: IL-6 first binds to the membrane-bound IL-6 receptor α subunit (mIL-6R) on target cells, forming an IL-6/IL-6R binary complex. This complex then recruits two molecules of the transmembrane signal transducer glycoprotein 130 (gp130), inducing their dimerization. As the intracellular domain of gp130 itself lacks kinase activity, its dimerized structure provides the physical platform for the associated Janus kinases (JAKs) to undergo mutual phosphorylation and activation. The activated JAKs subsequently phosphorylate specific tyrosine residues within the intracellular domain of gp130, thereby creating docking sites for signal transducers and activators of transcription, particularly STAT3. This ultimately triggers the phosphorylation, dimerization, and nuclear translocation of STAT3, and the transcription of its downstream target genes [98] (Figure 4). This elaborate cascade reaction constitutes the core framework of IL-6 classical signal transduction.

It is noteworthy that the expression of mIL-6R exhibits strict cell-type specificity. It is primarily confined to hepatocytes, certain leukocytes (e.g., macrophages, T cells), and muscle stem cells, among others [4]. Consequently, the scope of classical signaling is relatively limited. Its functions are typically associated with the hepatic acute phase response, immune cell differentiation, and specific tissue regeneration processes. In skeletal muscle, IL-6 secreted by muscle fibers themselves post-exercise likely acts primarily via classical signaling in an autocrine or paracrine manner on the fibers or neighboring satellite cells. This regulates glucose metabolism and promotes regenerative repair under physiological conditions. Thus, the classical signaling pathway serves as a key physiological route through which IL-6 contributes to maintaining skeletal muscle homeostasis and adaptive repair.

In contrast to the restricted scope of classical signaling, the trans-signaling pathway of IL-6 dramatically expands its potential target cell repertoire. It emerges as a key mechanism mediating widespread inflammation and tissue damage in pathological states. The core of this pathway lies in the existence of the soluble IL-6 receptor (sIL-6R). sIL-6R is generated mainly through proteolytic shedding (e.g., mediated by ADAM10/17) of the membrane receptor or via alternative splicing. In body fluids, the IL-6/sIL-6R complex can still bind to and activate cells expressing gp130, thereby “trans”-activating cells that do not express mIL-6R themselves [13]. This enables a vast array of cell types, including endothelial cells, smooth muscle cells, fibroblasts, and most epithelial cells, to respond to IL-6, significantly broadening the range of its pathological effects.

In the pathophysiology of skeletal muscle, trans-signaling is believed to play a crucial role, particularly in wasting diseases and chronic pathological conditions. For instance, in cancer cachexia, trans-signaling is one of the primary drivers of systemic inflammation and muscle atrophy [13]. In neuromuscular diseases or models of muscle denervation, abnormally activated interstitial cells, such as fibro-adipogenic progenitors (FAPs), secrete high levels of IL-6. This likely acts on surrounding myofibers and stromal cells via the trans-signaling pathway, thereby exacerbating muscle atrophy and fibrosis [59]. Further studies indicate that the IL-6/sIL-6R complex can induce a gene expression profile distinct from classical signaling in human airway smooth muscle cells and significantly promote cell proliferation, suggesting its unique role in pathological tissue remodeling [103]. These findings collectively highlight the potential value of targeting trans-signaling for intervening in muscle-wasting diseases.

Recent research has provided deeper insights into the regulatory details of the trans-signaling pathway, offering new avenues for its intervention. For example, cholesterol-rich lipid raft structures on the cell membrane provide a critical platform for the assembly of the IL-6/sIL-6R/gp130 signaling complex. The membrane trafficking process mediated by the EHD1 protein is essential for the construction and function of this signaling platform [104]. This suggests that targeting the assembly of the signaling complex or the membrane trafficking process may become a novel strategy for specifically inhibiting aberrant trans-signaling and mitigating pathological damage. Therefore, further elucidating the spatiotemporally specific regulatory networks of trans-signaling in different skeletal muscle disease models will be an important direction for future translational research.

### 4.2. Balance of the Two Signaling Pathways and Pathological Implications

Although classical and trans-signaling share the common downstream gp130-JAK-STAT3 core pathway, their ultimate biological effects can be markedly different. This divergence primarily stems from the cellular microenvironment of signal initiation, its kinetic features, and the specific gene expression profiles induced. For instance, during the development of obesity-related insulin resistance, classical IL-6 signaling within T cells promotes inflammation and insulin resistance in the early stage. In the late stage of obesity, classical signaling diminishes, while trans-signaling becomes significantly enhanced to sustain a chronic inflammatory state [105]. This dynamic shift from classical to trans-signaling profoundly reveals the temporal nature and complexity of IL-6’s mechanisms in disease progression. Therefore, understanding the regulatory mechanisms behind this signal switching is crucial for elucidating pathological processes.

In skeletal muscle pathophysiology, the balance between these two signaling modalities is decisive. Physiological, transient IL-6 release (e.g., post-exercise) primarily activates the classical signaling pathway, which confers metabolic protection and pro-regenerative functions [4]. Conversely, in chronic conditions such as cancer cachexia, amyotrophic lateral sclerosis, and age-related sarcopenia, persistently elevated systemic or local IL-6 levels are often accompanied by an increase in soluble IL-6 receptor (sIL-6R). This leads to the predominance of the trans-signaling pathway. Such an imbalance results in sustained STAT3 activation, upregulation of muscle atrophy-related genes (e.g., Atrogin-1, MuRF1), and stimulation of fibroblasts and immune cells. Ultimately, this cascade causes irreversible muscle mass loss and functional decline [27,59]. Recent research further emphasizes the importance of cell-specific signaling within local microenvironments. For example, during Langerhans cell antigen presentation, IL-6 trans-signaling to T cells is a key step in enhancing Th17 immune responses [106]. Moreover, quantitative analysis of IL-6 signaling components (e.g., IL-6, sIL-6R, sgp130) has shown potential for predicting disease severity and prognosis. In COVID-19 patients, a combination of these markers exhibits a powerful capacity for risk stratification [107]. Hence, precise dissection of the local signaling environment is a vital prerequisite for intervening in muscle pathologies.

#### Distinguishing Muscle-Derived Versus Immune-Derived IL-6

A critical distinction that is often overlooked in the literature is the cellular origin of IL-6. This distinction has profound implications for understanding its biological effects in skeletal muscle.

Muscle-derived IL-6 (myokine): Produced by contracting myofibers themselves in response to exercise, low glycogen, or calcium signaling [2,108]. Secreted rapidly (within minutes of contraction) and acts locally in an autocrine/paracrine manner. Typically associated with metabolic regulation (glucose uptake, lipolysis), satellite cell activation, and anti-inflammatory effects [4,57]. Exercise-induced muscle-derived IL-6 is not preceded by TNF-α or IL-1β elevation, distinguishing it from inflammatory IL-6 [2].

Immune-derived IL-6: Produced by infiltrating macrophages, T cells, and other immune cells in response to pathogens, damage-associated molecular patterns, or pro-inflammatory cytokines (TNF-α, IL-1β) [13,109]. Associated with chronic inflammation, cachexia, and pathological muscle wasting. Typically elevated in conditions such as cancer, sepsis, and aging [13,14].

Functional implications: The same IL-6 protein exerts different effects depending on its source and the accompanying cytokine milieu. Muscle-derived IL-6 during exercise promotes metabolic health and regeneration; immune-derived IL-6 in chronic disease drives atrophy and fibrosis [57]. Importantly, these sources are not mutually exclusive—in pathological states, both may contribute, and cross-talk between muscle and immune cells can amplify IL-6 signaling [13].

Throughout this review, we explicitly indicate the source of IL-6 (muscle-derived vs. immune-derived) when the literature allows; where studies do not distinguish, this limitation is noted.

### 4.3. Core Downstream Pathways

**Figure 5:**
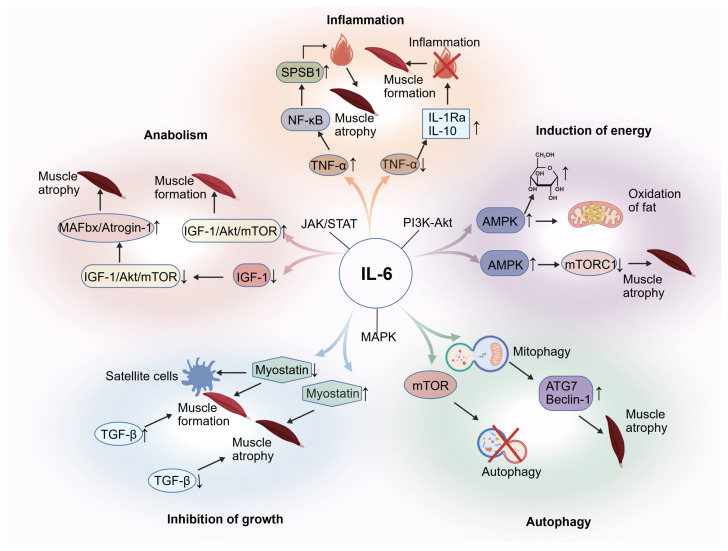
**The IL-6 signaling network and its integration with other key pathways in skeletal muscle.** IL-6 activates JAK/STAT, MAPK, and PI3K-Akt cascades, which interact extensively with anabolic (IGF-1/Akt/mTOR), inflammatory (TNF-α/NF-κB), growth-inhibitory (myostatin/TGF-β), energy-sensing (AMPK), and degradative (autophagy) pathways. This crosstalk determines the net effect of IL-6 on muscle mass and function. An upward arrow represents an upward expression, and a downward arrow represents a downward expression.

The signal transduction mechanisms of IL-6 exhibit a high degree of diversity and complexity. This directly dictates its capacity to exert strikingly different, and even opposing, effects in skeletal muscle physiology and pathology. Therefore, a thorough exploration of IL-6 signaling mechanisms is crucial for understanding its multifunctionality in the skeletal muscle pathophysiological process. Specifically, the diversity in IL-6 signaling arises not only from its initiation via two distinct modes—“classical signaling” and “trans-signaling”—but, more critically, from the networked crosstalk and dialog among its core downstream signaling pathways. Several key pathways, notably JAK/STAT, MAPK, and PI3K-Akt, are differentially activated and integrated depending on the specific physiological or pathological context (Figure 5). This precise regulation governs skeletal muscle metabolism, inflammation, regeneration, and atrophy [98]. The intricate interactions among these pathways present both opportunities and challenges for developing targeted therapies for skeletal muscle disorders.

#### 4.3.1. The JAK/STAT Pathway

The JAK/STAT pathway represents the most classical and characteristic signaling cascade for IL-6. Upon binding of IL-6 to the membrane-bound receptor IL-6Rα and gp130, gp130 dimerization is induced. This subsequently activates members of the Janus kinase (JAK) family, primarily JAK1, JAK2, and Tyk2, which are constitutively associated with the intracellular domains of gp130. The activated JAKs phosphorylate specific tyrosine residues within the cytoplasmic region of gp130, creating docking sites for STAT proteins, particularly STAT3. Following recruitment and phosphorylation by JAKs, STAT3 forms homo- or heterodimers. These dimers then translocate into the nucleus, where they function as transcription factors to regulate the expression of a series of target genes. These include BCL-2 (involved in cell survival), c-Myc (associated with proliferation), and the crucial negative feedback regulator SOCS3 [27]. In skeletal muscle pathologies, constitutively activated STAT3 serves as a core transcriptional regulator driving muscle atrophy. For instance, in denervation models, fibro/adipogenic progenitors (FAPs) within the muscle interstitium exhibit aberrant, persistent STAT3 activation. These FAPs secrete high levels of IL-6, which then acts on myofibers in a paracrine manner. This signaling upregulates the expression of key atrophy-related genes, Atrogin-1 and MuRF1, ultimately leading to myofiber atrophy and fibrosis [59]. Further studies reveal that the transcriptional activity of STAT3 is not solely regulated by its phosphorylation status. It is also finely modulated by its binding to specific genomic enhancer regions, epigenetic modifications, and interactions with other transcription factors. This complexity partially explains how IL-6 signaling can produce distinct biological effects in different cell types and microenvironments [98]. Notably, the trans-signaling pathway is considered the primary mode driving sustained STAT3 activation and pathological responses.

#### 4.3.2. The MAPK Pathway

Beyond the JAK/STAT pathway, IL-6 can also potently activate several members of the mitogen-activated protein kinase (MAPK) family. These include extracellular signal-regulated kinase 1/2 (ERK1/2), p38 MAPK, and c-Jun N-terminal kinase (JNK). Activation of these pathways is typically initiated by gp130. It recruits SHP2 (PTPN11), which in turn adapts growth factor receptor-bound protein 2 (Grb2) and the guanine nucleotide exchange factor SOS. This triggers the Ras-Raf-MEK-ERK cascade. Concurrently, gp130 can also activate the p38 MAPK and JNK pathways [104]. In skeletal muscle, the MAPK pathways are involved in regulating myocyte proliferation, differentiation, stress responses, and the generation of inflammatory mediators. For instance, ERK1/2 activation is often associated with pro-survival and proliferative signals. In contrast, p38 MAPK and JNK primarily respond to stress and inflammatory stimuli. They participate in inducing the expression of various inflammatory factors, including IL-6 itself. This creates a positive feedback loop that amplifies the inflammatory response [99]. Such a positive feedback mechanism may play a significant role in muscle wasting associated with chronic low-grade inflammation. The spatiotemporal activation patterns of different MAPK branches add another layer of complexity to how IL-6 signaling regulates muscle homeostasis and pathology. Furthermore, extensive crosstalk exists between the MAPK and JAK/STAT pathways. Together, they shape the cellular transcriptomic response.

#### 4.3.3. The PI3K-Akt Pathway

IL-6 signaling can also phosphorylate and activate phosphatidylinositol 3-kinase (PI3K) and its key downstream effector, protein kinase B (Akt). The activation of this pathway may occur through various mechanisms, including the direct phosphorylation of insulin receptor substrate proteins by JAK or SHP2, which subsequently recruit and activate PI3K. Under physiological conditions such as exercise, myogenic IL-6, secreted by skeletal muscle itself, exerts crucial beneficial metabolic regulatory effects by activating the PI3K-Akt pathway. Activated Akt promotes the translocation of glucose transporter 4 (GLUT4) to the cell membrane, enhances glycogen synthesis, and improves systemic insulin sensitivity [4]. Concurrently, Akt provides strong survival signals to muscle cells by phosphorylating and inhibiting pro-apoptotic proteins such as Bad and FoxO transcription factors. However, under pathological conditions, the pro-inflammatory signaling of IL-6, potentially in concert with other factors, can disrupt the normal feedback balance of PI3K-Akt signaling, leading to insulin resistance and metabolic disorders. Recent studies also suggest that the trans-signaling of IL-6 may be more inclined to activate pro-inflammatory pathways, whereas its classic signaling is associated with certain metabolic benefits. This pathway preference adds complexity to the interpretation of IL-6’s functions [110]. Notably, Akt can also phosphorylate and inhibit glycogen synthase kinase-3β (GSK-3β), indirectly affecting the activity of various transcription factors, including STAT3, thereby forming a complex inter-pathway regulatory network.

In summary, the downstream signaling of IL-6 does not operate in isolation but constitutes a highly interconnected network. Extensive cross-talk exists among the JAK/STAT, MAPK, and PI3K-Akt pathways. For instance, STAT3 activity can be regulated by MAPK, while SOCS3, as a feedback inhibitor of the JAK/STAT pathway, can also influence other pathways. The diversity and spatiotemporal specificity of these signaling pathways, along with their complex interactions, collectively determine the dual role of IL-6 in skeletal muscle. It acts both as a ‘messenger’ for exercise adaptation and metabolic health and can transform into a ‘driver’ of atrophy and fibrosis [98]. Understanding the activation patterns and integrative mechanisms of these core pathways within specific cellular contexts is key to deciphering the dual role of IL-6 in skeletal muscle pathophysiology. This understanding also provides a precise molecular map for targeted interventions.

### 4.4. Negative Feedback Regulation

The suppressor of cytokine signaling (SOCS) family, particularly SOCS3, serves as a core negative feedback regulator of IL-6 signaling. Its expression is directly induced by activated STAT3, forming a classic “activation-inhibition” loop. The SOCS3 protein functions primarily through two mechanisms. Firstly, its SH2 domain binds to phosphorylated gp130 or JAK kinases. It then utilizes its “kinase inhibitory region” to directly inhibit the catalytic activity of JAK1/JAK2/TYK2. Secondly, it acts as an adaptor for an E3 ubiquitin ligase complex. This recruits the receptor complex and promotes its degradation via the proteasome pathway, thereby rapidly terminating signal transduction [98,110]. Thus, through multi-layered and precise intervention, SOCS3 ensures that IL-6 signaling is promptly and effectively constrained under physiological conditions.

Beyond SOCS proteins, other molecules participate in this intricate negative regulatory network. For instance, the SH2 domain-containing protein tyrosine phosphatase 2 (SHP2) can be recruited to phosphorylated gp130. It then negatively regulates the JAK-STAT pathway through dephosphorylation [98]. Simultaneously, activated STAT proteins can induce the expression of protein inhibitors of activated STAT (PIAS) family proteins. These suppress transcriptional activity by blocking STAT dimer binding to DNA or promoting their SUMOylation [110]. Together, these mechanisms constitute a multi-step, collaborative post-transcriptional regulatory network. This network profoundly influences the intensity and duration of IL-6 signaling.

In pathological states, dysregulation of these negative feedback mechanisms often leads to serious consequences. For example, insufficient expression or functional deficiency of SOCS3 can cause sustained activation of IL-6 signaling. This drives chronic inflammation and exacerbates tissue damage [111]. Conversely, in certain chronic disease contexts, overexpression of SOCS3 may lead to cellular desensitization to IL-6. This subsequently impairs its beneficial roles in maintaining metabolic homeostasis and tissue repair [105]. Research in muscle denervation models provides an illustration: the abnormally sustained activation of STAT3–IL-6 signaling in fibro-adipogenic progenitors (FAPs) may be linked to impaired local SOCS3 feedback. This ultimately drives muscle atrophy and fibrosis [59]. This indicates that the precise balance of negative feedback pathways is crucial for maintaining skeletal muscle homeostasis.

The complexity of the IL-6 signaling pathway is also reflected in the potentially differential negative feedback regulation of its “classical signaling” and “trans-signaling” modes. Here, soluble gp130 (sgp130) acts as a natural antagonist of the IL-6/sIL-6R complex. It can specifically inhibit trans-signaling while having minimal impact on classical signaling. This provides an important specific target for intervention [107,110]. Furthermore, cellular microenvironment factors (such as lipid raft composition and epigenetic modifications) may also influence the expression and function of regulatory components like SOCS. This ultimately determines the biological effects of IL-6 signaling [98]. Therefore, in-depth analysis of the spatiotemporal specificity of these negative feedback networks and their disturbance under pathological conditions is key. It is essential for elucidating the dual role of IL-6 in skeletal muscle and for developing targeted therapies.

### 4.5. Synthesis and Commentary

Section 4 dissects the molecular machinery of IL 6 signal transduction, emphasizing the dichotomy between classical and trans signaling, the hierarchy of downstream pathways, and negative feedback regulation. Key insights include the following: Classical signaling (mIL 6R/gp130) is cell type restricted and generally associated with homeostatic functions (metabolic regulation, regeneration); trans signaling (sIL 6R/gp130) vastly expands the range of responsive cells and is the dominant mode in chronic inflammation and cachexia [13,57]. The JAK/STAT3 axis is the core effector pathway for both modes, while MAPK and PI3K Akt serve modulatory roles [4,100]. Negative feedback via SOCS3 and sgp130 is essential for terminating signals and preventing pathological overactivation.

Major unresolved questions include: (1) How is the classical vs. trans signaling balance regulated in specific muscle diseases? The mechanisms that determine whether IL 6 signals via mIL 6R or sIL 6R are poorly understood, but may involve ADAM10/17-mediated shedding of mIL 6R or alternative splicing. (2) Can we therapeutically target trans signaling without affecting classical signaling? sgp130Fc (olamkicept) is a promising tool, but its efficacy in muscle wasting has not been tested in humans [112]. (3) What determines pathway preference (STAT3 vs. MAPK vs. Akt) in different contexts? Post-translational modifications, receptor clustering, and lipid raft composition may play a role, but systematic studies are lacking. (4) Why does STAT3 activation sometimes promote regeneration (post-exercise) and other times drive atrophy (cachexia)? The answer likely lies in signal duration, amplitude, and cooperating transcription factors (e.g., NF κB, AP 1). Single-cell and spatial transcriptomics could resolve these context-specific regulatory codes.

## 5. Upstream Mechanisms Regulating IL-6 Expression and Function

**Figure 6:**
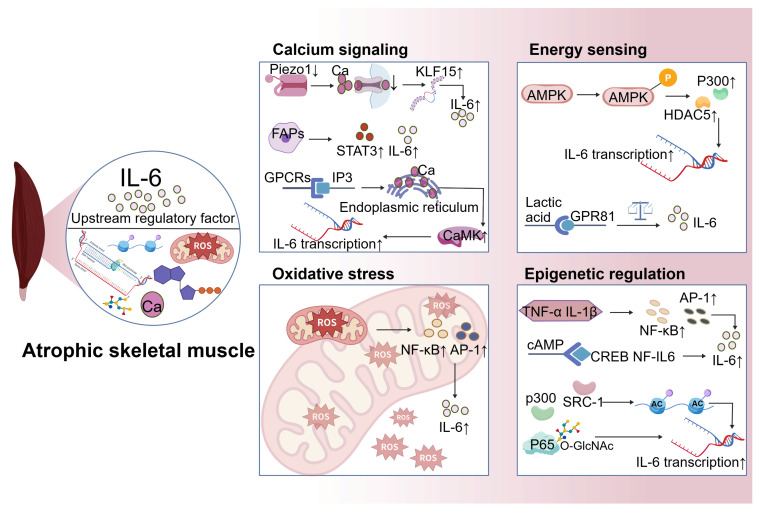
**Key upstream mechanisms regulating IL-6 expression in skeletal muscle.** IL-6 production is controlled by multiple inputs: (1) reduced mechanical stimulation decreases Piezo1-mediated calcium influx, upregulating KLF15 and IL-6; (2) energy stress activates AMPK, promoting IL-6 transcription; (3) mitochondrial ROS activate NF-κB/AP-1, enhancing IL-6 expression; (4) epigenetic modifications (e.g., histone acetylation, O-GlcNAcylation) fine-tune IL-6 gene accessibility. An upward arrow represents an upward expression, and a downward arrow represents a downward expression.

The expression and function of IL-6 in skeletal muscle are regulated by a multilayered and finely tuned upstream signaling network. These signals primarily arise from mechanical, metabolic, and redox changes experienced by the muscle. Ultimately, they influence IL-6 gene transcription and protein function through transcription factors and epigenetic modifications (Figure 6). A thorough exploration of these upstream mechanisms is key to understanding the dichotomous roles of IL-6 in muscle physiology and pathology.

### 5.1. Calcium Signaling and Mechanotransduction

Intracellular calcium ions (Ca^2+^) serve as a crucial second messenger, linking mechanical muscle activity to gene expression. Mechanosensitive Piezo1 ion channels play a significant role in maintaining the basal cytosolic calcium concentration ([Ca^2+^]i). Muscle disuse or denervation downregulates Piezo1 channel expression. The subsequent decrease in [Ca^2+^]i is an upstream event that induces the expression of KLF15 and IL-6, constituting one of the early signaling pathways in muscle atrophy [26]. Furthermore, in denervation-activated fibro-adipogenic progenitors (FAPs), sustained STAT3-IL-6 signaling exacerbates muscle fiber atrophy and fibrosis. This suggests that the loss of mechanical signals may amplify the pathological role of IL-6 through non-autonomous cellular mechanisms [58,59]. Conversely, during active muscle contraction, IP3-mediated endoplasmic reticulum calcium release—triggered by excitation-contraction coupling or G protein-coupled receptor activation—is a key mechanism for the rapid transcription of the IL-6 gene [113]. These contraction-dependent calcium transients likely regulate the activity of transcription factors such as NFAT and CREB. This regulation occurs through the activation of downstream effectors like calcineurin or Ca^2+^/calmodulin-dependent protein kinases (CaMK). Ultimately, this process initiates the synthesis and release of IL-6.

### 5.2. Energy Sensing and Metabolic Stress

AMP-activated protein kinase (AMPK) serves as a cellular energy sensor. It is activated in skeletal muscle under energy-stress conditions, such as exercise or low glycogen levels [34,114]. Activated AMPK promotes IL-6 expression through multiple mechanisms. On one hand, it can directly phosphorylate and activate transcriptional co-regulators like HDAC5 and p300, thereby enhancing IL-6 gene transcription [115]. On the other hand, it may also increase IL-6 protein synthesis by stabilizing IL-6 mRNA. Thus, AMPK plays a central role in regulating muscular IL-6 production. Notably, AMPK activation also exhibits anti-inflammatory regulatory capacity. For instance, pharmacological activation of AMPK in the liver can inhibit the phosphorylation of JAK1 and STAT3, downstream components of IL-6 signaling [116]. This suggests that AMPK holds bidirectional regulatory potential over the IL-6 signaling network. This context-dependent regulatory mode provides crucial clues for understanding the complex role of IL-6 at the intersection of metabolism and inflammation.

Beyond AMPK, lactate—a metabolic byproduct of exercise—is also recognized as a significant signaling molecule that induces IL-6 production. Lactate can indirectly influence IL-6 expression levels through its receptor GPR81 or by modulating intracellular acidification and redox balance. This indicates a multi-layered interaction between metabolic signals and cytokine release during exercise physiology. Recent research has further revealed the feedback regulatory role of IL-6 signaling itself in systemic energy metabolism. In individuals with type 2 diabetes or obesity, IL-6 signaling is involved in regulating the secretion of glucagon-like peptide-1 (GLP-1). Blocking the IL-6 receptor reduces exercise-induced GLP-1 levels, thereby establishing a bidirectional communication loop between muscle and the endocrine system [111]. The discovery of this muscle-endocrine axis highlights the pivotal position of IL-6 in integrating metabolic adaptation and physiological responses.

### 5.3. Oxidative Stress and Mitochondrial Function

Mitochondria serve as the cellular powerhouses and are also a primary source of reactive oxygen species (ROS). Their functional status is closely associated with IL-6-mediated muscle atrophy [117,118]. In systemic inflammatory conditions, such as sepsis, IL-6 can inhibit the expression of peroxisome proliferator-activated receptor gamma coactivator 1-alpha (PGC-1α). This reduces mitochondrial biogenesis and increases ROS production. Subsequently, it activates both the ubiquitin-proteasome system and the autophagy-lysosome pathway, ultimately accelerating protein degradation [14,119]. Conversely, IL-6 gene deficiency can upregulate PGC-1α and suppress ROS generation, thereby exerting a muscle-protective effect. This suggests that targeting the IL-6/PGC-1α axis may be a potential strategy to alleviate inflammatory muscle atrophy. In cancer cachexia models, IL-6 signaling can induce increased expression of dynamin-related protein 1 (DRP-1) and mitochondrial fission 1 protein (FIS-1). This leads to mitochondrial network fragmentation, decreased membrane potential, and impaired ATP synthesis, thereby exacerbating muscle wasting [100]. The imbalance in mitochondrial fission and fusion dynamics is now considered a key link connecting inflammatory signaling to muscle metabolic dysfunction. Furthermore, mitochondrially derived ROS themselves can act as signaling molecules. They activate pro-inflammatory transcription factors such as NF-κB and AP-1, which further amplify the expression of cytokines like IL-6. Beyond NF-κB and AP-1, mitochondrial DNA (mtDNA) released into the cytoplasm under oxidative stress serves as a potent endogenous danger signal that activates the cGAS-STING pathway. Emerging evidence indicates that aberrant cGAS-STING activation in skeletal muscle contributes to chronic inflammation, metabolic dysfunction, and muscle atrophy, establishing this pathway as a central regulator in muscle pathophysiology [120]. Thus, mtDNA-driven cGAS-STING signaling may represent an additional layer of upstream control over IL-6 expression in conditions such as sepsis, aging, and cachexia. This creates a positive feedback loop that continuously promotes muscle atrophy [121]. Therefore, a deeper analysis of the bidirectional regulatory network between IL-6 and mitochondria will provide an important theoretical basis for intervening in muscle atrophy.

### 5.4. Epigenetic and Transcriptional Factor Regulation

The transcription of the IL-6 gene is under strict spatiotemporal control, a process dependent on the coordinated action of multiple transcription factors and epigenetic modifications. Among these, classical pro-inflammatory transcription factors such as nuclear factor-κB (NF-κB) and activator protein-1 (AP-1), activated by stimuli like TNF-α and IL-1β, serve as major drivers for inducing IL-6 expression. Furthermore, cAMP response element-binding protein (CREB) and NF-IL6 (C/EBPβ) also actively participate in regulating the IL-6 gene [122]. Thus, a complex network of transcription factors collectively ensures the precise expression of IL-6 under appropriate physiological or pathological conditions.

Epigenetic modifications provide another layer of fine control over IL-6 transcription. For instance, in vascular smooth muscle cells, angiotensin II-induced IL-6 expression requires the involvement of the histone acetyltransferase p300 and steroid receptor coactivator-1 (SRC-1). These enzymes acetylate histones to loosen chromatin structure, thereby facilitating the assembly of transcriptional complexes [122]. Beyond histone modifications, post-translational protein modifications such as O-GlcNAcylation are increasingly recognized. Studies indicate that cold exposure stress can upregulate IL-6 expression by enhancing O-GlcNAcylation of the transcription factor p65, promoting its activity and nuclear translocation [27]. These epigenetic mechanisms further enrich the regulatory hierarchy of IL-6, enabling it to respond to diverse environmental signals.

On the other hand, as a core downstream effector molecule of IL-6 signaling, the activity of STAT3 itself is also subject to precise epigenetic and transcriptional regulation. Notably, sustained STAT3 activation can establish a positive feedback loop and synergize with factors like NF-κB, thereby maintaining a high-expression state of IL-6. This phenomenon is particularly prominent in chronic inflammation and cancer cachexia [98]. Such a positive feedback mechanism highlights the self-sustaining nature of IL-6 signaling under pathological conditions, offering potential targets for intervention.

Recent studies also reveal that viral oncoproteins (e.g., HPV E6) can drive IL-6 autocrine signaling by activating the Rac1-NF-κB axis, which subsequently activates STAT3. This provides a new perspective for understanding the aberrant persistence of IL-6 expression in pathological settings [123]. Consequently, virus-mediated activation of IL-6 signaling may play a key role in the development of certain cancers. Furthermore, single-nucleotide polymorphisms (SNPs) at specific gene loci, such as rs2228145 in the IL-6 receptor (IL6R), can influence the intensity of classical IL-6 signal transduction and are closely associated with the risk and therapeutic response of various inflammatory diseases. This underscores the complexity of IL-6 pathway regulation from a genetic standpoint [124]. In summary, the transcriptional regulation of IL-6 is a multidimensional and finely tuned process, involving a variety of mechanisms including transcription factors, epigenetic modifications, feedback loops, and genetic variations.

### 5.5. Synthesis and Commentary

Section 5 outlines the upstream mechanisms that control IL-6 expression and function in skeletal muscle, spanning mechanotransduction (Piezo1/Ca^2+^/KLF15), energy sensing (AMPK, lactate), oxidative stress (mitochondrial ROS), and epigenetic regulation (histone acetylation, O-GlcNAcylation). A landmark finding is the Piezo1/KLF15/IL-6 axis in disuse atrophy: reduced mechanical load → decreased Piezo1 → lowered basal [Ca^2+^]i → KLF15 upregulation → IL-6 transcription [26]. This represents a new paradigm whereby a decrease in Ca^2+^ from baseline (rather than an increase) acts as a signaling event. Another key insight is the bidirectional relationship between IL-6 and mitochondria: IL-6 suppresses PGC-1α, leading to mitochondrial ROS production, which in turn activates NF-κB to further enhance IL-6 expression, creating a positive feedback loop [14].

Critical knowledge gaps remain: (1) How do mechanical signals (Piezo1) integrate with metabolic signals (AMPK) to fine-tune IL 6 expression? It is unknown whether these pathways converge on the same transcription factors or operate in parallel. (2) Are there muscle fiber-type-specific differences in Piezo1 expression or Ca^2+^ handling that explain the higher IL 6 secretory capacity of slow-twitch fibers? [25]. (3) Can epigenetic modifiers (e.g., HDAC inhibitors, OGT modulators) be leveraged to selectively enhance beneficial IL 6 expression (post exercise) or suppress pathological expression (cachexia)? Preliminary studies with cold exposure and O GlcNAcylation suggest promise [27], but off-target effects are a major concern. (4) The role of non-coding RNAs (e.g., miR 497 5p) in regulating IL 6 expression is just emerging [54]; systematic mapping of the IL 6-related miRNA network in different muscle conditions is needed.

## 6. Challenges and Controversies

Despite significant progress in understanding the role of IL-6 in skeletal muscle pathophysiology, translating this knowledge into effective clinical intervention strategies still faces multiple fundamental challenges and controversies. These challenges primarily stem from the extreme complexity of the IL-6 signaling network, the context-dependent nature of its effects, and the substantial gap between basic research findings and their application to human diseases.

### 6.1. Context-Dependency of Signaling Pathways

The most remarkable feature of IL-6 in skeletal muscle biology is the high degree of context-dependency in its function. Rather than a simple ‘good versus bad’ dichotomy, IL-6’s effects are determined by the integration of four key parameters: (1) context—physiological (exercise) versus pathological (cancer, aging); (2) concentration—acute low-level (pM) versus chronically elevated (nM); (3) duration—transient pulses versus sustained exposure; and (4) signaling topology—classical (mIL-6R-dependent) versus trans-signaling (sIL-6R-dependent). Its acute and transient increase typically promotes beneficial metabolic adaptations and tissue repair. Conversely, when chronically and persistently elevated (as commonly seen in cancer, aging, and chronic inflammatory diseases), it drives muscle wasting and dysfunction. The precise molecular mechanisms that integrate these four parameters remain incompletely elucidated, constituting a central scientific challenge for current mechanistic research and the development of targeted therapeutic strategies. Therefore, deciphering the precise conditions that determine the functional direction of IL-6 is a primary challenge for translational research.

First, the diversity of IL-6 signaling pathways forms the structural basis for its functional context-dependency. IL-6 activates “classical signaling” by binding to the membrane-bound IL-6 receptor (IL-6R). This pathway is typically restricted to specific cell types, such as hepatocytes and certain leukocytes, and is often associated with anti-inflammatory and tissue-protective effects. However, in most cells lacking membrane IL-6R (like myofibers and adipocytes), IL-6 must first form a complex with the soluble IL-6 receptor (sIL-6R). This complex then activates the ubiquitously expressed membrane protein gp130, initiating “trans-signaling.” Substantial evidence indicates that muscle atrophy in chronic pathological states relies more heavily on trans-signaling. For example, in a pancreatic cancer cachexia model, tumor cells induce IL-6 production from adipocytes, while muscle cells express sIL-6R. This creates a feed-forward IL-6 trans-signaling loop that drives lipolysis and muscle wasting [13]. This suggests that distinguishing and selectively inhibiting pathological trans-signaling, while preserving physiological classical signaling, represents a major challenge and a potential breakthrough for future drug design.

Second, the spatiotemporal dynamics of IL-6 downstream signaling and its complex crosstalk with effector networks collectively determine its ultimate biological outcome in skeletal muscle. After acute exercise, IL-6 secreted autocrinely by skeletal muscle primarily activates pathways like AMPK and PI3K/Akt. This enhances glucose uptake and insulin sensitivity, and may promote metabolic health by modulating autophagy and mitochondrial biogenesis [4]. In contrast, under conditions like the tumor microenvironment or aging, chronic IL-6 exposure tends to persistently activate the JAK/STAT3 pathway. This upregulates the expression of E3 ubiquitin ligases such as muscle atrophy F-box protein (MAFbx/Atrogin-1) and muscle RING-finger protein-1 (MuRF1). It may also suppress the anabolic IGF-1/Akt/mTOR pathway. These changes ultimately lead to increased net protein catabolism [8]. This shift in signaling preference may involve deeper epigenetic reprogramming. For instance, studies show that IL-6 can suppress the expression of peroxisome proliferator-activated receptor γ coactivator-1α (PGC-1α) via a STAT3-mediated mechanism. This promotes mitochondrial reactive oxygen species (ROS) production and exacerbates sepsis-induced muscle atrophy [14]. Furthermore, the interplay between IL-6 and other cytokines (e.g., TNF-α, IL-1β) can generate synergistic or antagonistic effects, adding another layer of context-specificity to its function. Therefore, a systematic understanding of the dynamic network changes at the IL-6 signaling hub under different pathophysiological contexts is a prerequisite for accurately predicting and effectively intervening in its pathological effects.

### 6.2. Cellular Specificity and Tissue Heterogeneity

Skeletal muscle is not a homogeneous organ. The cellular heterogeneity within it and between other tissues profoundly influences the biological effects of IL-6. This presents a significant challenge for developing precisely targeted therapies. At the myofiber level, different fiber types exhibit marked differences in their secretion of and response to IL-6. For instance, slow-oxidative muscle fibers (e.g., soleus) demonstrate a greater capacity for IL-6 secretion under various stimuli compared to fast-glycolytic fibers (e.g., extensor digitorum longus) [25]. This disparity may stem from differences in metabolic profiles, calcium handling capabilities, and transcription factor repertoires. For example, Piezo1-mediated downregulation of calcium signaling can induce Krüppel-like factor 15 (KLF15) and IL-6 expression, thereby mediating disuse-induced muscle atrophy. The activity of this signaling axis likely varies across different fiber types [26]. Furthermore, the regulation of systemic metabolism by muscle-derived IL-6 shows sexual dimorphism, indicating an important interaction between hormonal milieu and IL-6 signaling [7]. Therefore, understanding fiber-type-specific responses is crucial for designing targeted interventions.

Beyond myofibers, other cell populations within skeletal muscle also play key roles in IL-6 signaling. Satellite cells, the stem cells for muscle regeneration, have their functions dynamically modulated by IL-6 levels. Low levels of IL-6 can promote their proliferation and differentiation, whereas chronically elevated IL-6 suppresses their function. It may even induce them to secrete senescence-associated secretory phenotype (SASP) factors, such as IL-6 itself and osteopontin (SPP1). This, in turn, can drive the expansion of fibro-adipogenic progenitors (FAPs) and promote fibrosis [125]. Concurrently, immune cells like macrophages infiltrate muscle extensively during injury or pathology. They are both a significant source of IL-6 and a target of its action. The plasticity of macrophage polarization is critically regulated by the cGAS-STING pathway, which senses cytosolic DNA and drives M1-biased pro-inflammatory responses. Activation of cGAS-STING in macrophages promotes the secretion of IL-6 and other inflammatory cytokines, thereby amplifying the local inflammatory milieu within skeletal muscle [126]. Conversely, defective cGAS-STING signaling may impair macrophage-mediated tissue repair, highlighting its dual role in modulating IL-6 production and muscle inflammation. IL-6 can influence macrophage phenotype polarization (pro-inflammatory M1 vs. repair-promoting M2), which subsequently regulates the muscle repair process [53]. Additionally, adipocytes and muscle cells engage in “crosstalk” via factors like IL-6. In pathological states such as cachexia, this dialogue becomes dysregulated, leading to enhanced lipolysis and muscle metabolic disturbance [13]. These intricate cellular interactions form the regulatory network of IL-6 within the muscle microenvironment.

Tissue-level heterogeneity is equally critical. Muscle-derived IL-6 can act as a “myokine” on distant organs like the liver, adipose tissue, brain, and even bone, thereby modulating systemic energy homeostasis. For example, IL-6 released from muscle post-exercise enhances hepatic glucose output and adipose tissue lipolysis. However, studies on muscle-specific IL-6 knockout mice show that its regulatory effect on hepatic glucose metabolism differs markedly between exercise and resting states [44,127]. This long-distance effect implies that interventions targeting muscle IL-6 may trigger unforeseen metabolic chain reactions throughout the body. Consequently, the multi-organ action profile of IL-6 must be systematically considered when designing therapeutic strategies.

In summary, the multi-layered heterogeneity of skeletal muscle cells and tissues collectively shapes the complex biological effects of IL-6 signaling. Future research needs to further dissect its precise mechanisms of action within specific cell types and pathological contexts. This is essential to overcome the current challenges facing targeted therapies.

### 6.3. Discrepancies Between Animal Models and Human Diseases

Current understanding of the role of IL-6 in skeletal muscle pathophysiology relies heavily on studies using genetically engineered mouse models. These include systemic or conditional IL-6 knockout mice, mdx dystrophy mice, and tumor-transplant cachexia models. While indispensable, these models have inherent limitations. Their differences from complex human chronic diseases hinder the translation of research findings into clinical practice. Therefore, conclusions drawn from animal experiments must be interpreted with caution when applied to humans.

A particularly important limitation is the use of supraphysiological IL-6 concentrations in many preclinical studies. While human physiological IL-6 levels range from 1–5 pg/mL at rest and peak at 50–200 pg/mL during intense exercise [2], many in vitro and in vivo studies use concentrations in the ng/mL range (100–1000-fold higher than physiological levels). For example, studies reporting direct atrophic effects of IL-6 on myotubes often use 100 ng/mL recombinant IL-6 [19,100]—a concentration that may never be achieved in human skeletal muscle interstitium under physiological or even most pathological conditions. This dose discrepancy raises important questions about whether observed effects represent true physiological/pathophysiological mechanisms or pharmacological artifacts. Exceptions include studies using IL-6 concentrations in the pM range (approximately 2–20 pg/mL) that more closely approximate human physiology [4]. Future studies should explicitly justify their choice of IL-6 concentration relative to known human or mouse physiological ranges, and researchers should exercise caution when extrapolating from supraphysiological dose studies.

Duchenne muscular dystrophy (DMD) serves as a prime example. In mdx mice (a model of DMD), early studies indicated elevated IL-6 levels. Treatment with the anti-IL-6 receptor antibody (MR16-1) showed beneficial effects in some studies, promoting muscle regeneration and reducing fibrosis [29]. However, the disease progression in human DMD patients is slower and more complex. Their inflammatory networks exhibit greater redundancy. It remains uncertain whether targeting IL-6 alone in clinical trials can replicate the efficacy observed in animal models. In fact, a recent study investigating the effect of the IL-1β inhibitor canakinumab on IL-6 secretion in human myotubes suggested that multi-target anti-inflammatory strategies might be necessary for DMD treatment [128]. This indicates that for complex diseases, blocking a single inflammatory factor may be insufficient to achieve ideal clinical outcomes.

In cancer cachexia research, commonly used mouse models like colon adenocarcinoma (C26) or Lewis lung carcinoma (LLC) transplants rapidly induce severe muscle and fat wasting. This is accompanied by a marked increase in inflammatory factors, including IL-6. However, human cancer cachexia is a highly heterogeneous, multi-stage process. Not all patients exhibit significant systemic inflammation, and their inflammatory cytokine profiles are likely more complex. For instance, blocking IL-6 signaling improves muscle regeneration in mdx mice. Yet, in a more severe dystrophy model (dystrophin/utrophin double knockout mice), IL-6 receptor antibody improved limb muscle pathology but failed to alleviate degeneration in the diaphragm and heart [29]. This highlights the specific impact of disease stage and muscle type on therapeutic response. Furthermore, animal models typically use young, genetically homogeneous individuals. Human patients, however, present with multiple confounding factors. These include aging, comorbidities, and medication history, all of which can significantly influence the responsiveness of the IL-6 signaling pathway. Therefore, the simplified systems of animal models cannot fully replicate the complex internal environment of human disease.

In summary, extrapolating findings from animal models to clinical applications in humans requires extreme caution. Future research needs to actively utilize more physiologically relevant in vitro models. These include muscle organoids and muscle-on-a-chip systems [129], which better approximate the human physiological environment. This approach should be combined with multi-omics analyses of patient serum and muscle biopsy samples. Such integration is necessary to validate related mechanisms and re-evaluate the relevance of IL-6 as a therapeutic target in the context of human disease. By consolidating data from diverse research models and clinical sources, a solid foundation can be laid for developing effective intervention strategies.

### 6.4. Timing of Intervention

Given the coexistence of IL-6’s physiological and pathological effects, complete suppression of its signaling may be a “double-edged sword.” One of the greatest therapeutic challenges is achieving selective inhibition in terms of timing and functional dimensions. The primary risk lies in impairing normal muscle regeneration and metabolic health. The acute increase in IL-6 induced by exercise is crucial for activating satellite cells, enhancing muscle glucose uptake, and promoting hepatic glucose output. Using IL-6 pathway inhibitors, such as tocilizumab, in healthy individuals may compromise exercise adaptation and affect metabolic regulation. A human trial found that while IL-6R blockade altered fatty acid metabolism during the post-exercise recovery period, it did not affect muscle glycogen resynthesis, suggesting selective impacts on different metabolic pathways [130]. However, the potential adverse effects of long-term blockade in pathological states still require comprehensive evaluation. For instance, during the early stages of infection or tissue injury, IL-6 is vital for initiating immune defense and tissue repair; inhibition at this stage may increase infection risk or delay recovery.

Secondly, the timing of intervention must be based on the dynamic progression of the disease. In cancer cachexia, muscle wasting may begin early in the disease, but clinical manifestations often appear only in advanced stages. Should preventive intervention be applied during the pre-cachexia stage, or treatment during the overt stage? The dominant role of IL-6 may differ across stages. Research in tumor-bearing mice showed that the MEK inhibitor selumetinib reduced tumor burden and IL-6 levels, yet failed to prevent muscle atrophy and potentially exacerbated weight loss [131]. This indicates that merely lowering IL-6 levels may be insufficient to reverse established wasting programs or may trigger other compensatory catabolic pathways.

Finally, strategies for achieving functional selectivity are still under exploration. One approach is developing biased ligands or allosteric modulators to selectively activate beneficial downstream pathways, such as MAPK, while inhibiting harmful ones, such as STAT3. Another strategy involves using specific inhibitors of trans-signaling, such as sIL-6R neutralizers or sgp130Fc (e.g., olamkicept). These aim to selectively block pathological trans-signaling while preserving classical anti-inflammatory signaling. Additionally, targeting more specific downstream effectors of IL-6 signaling, like particular E3 ubiquitin ligases, rather than the upstream cytokine itself, is considered a potentially side-effect-reducing strategy. However, all these approaches require meticulous testing of timing and dosage in complex human disease contexts. The goal is to find the optimal intervention point within the delicate balance between “pro-hypertrophy” and “pro-atrophy,” as well as “pro-inflammatory” and “anti-inflammatory” signals.

In summary, IL-6 plays a complex, context-dependent, and central role in skeletal muscle. Overcoming the aforementioned challenges requires interdisciplinary efforts, integrating research from molecular and cell biology to clinical medicine. Through innovative models and precise clinical trial designs, this multifaceted cytokine can ultimately be transformed into a powerful tool for improving muscle health.

### 6.5. Synthesis and Commentary

Section 6 addresses the major challenges and controversies that impede translation of basic IL 6 research into clinical practice. These include: context dependency (signaling pathway, concentration, duration, cellular source); cellular and tissue heterogeneity (myofibre types, satellite cells, FAPs, immune cells); animal–human discrepancies (supraphysiological IL 6 doses, simplified genetics, lack of comorbidities); and timing of intervention (preserving beneficial IL 6 while blocking pathological effects).

A key controversy highlighted is whether IL 6 is a valid therapeutic target in human muscle wasting given that most preclinical success comes from models with extreme IL 6 elevations (e.g., C26 tumors). In human cancer cachexia, IL 6 levels are often only modestly elevated, and anti-IL 6 trials have shown limited efficacy (e.g., in non-small-cell lung cancer). This suggests that IL 6 may be a necessary but not sufficient driver, and that redundant pathways (TNF α, myostatin, activin) may compensate when IL 6 is blocked. Another major challenge is the risk of impairing exercise-induced metabolic benefits with systemic IL 6 blockade. Tocilizumab is increasingly used in rheumatoid arthritis, yet its long-term effects on muscle health and physical function remain understudied.

Future research must: (1) Develop humanized or patient-derived xenograft models that recapitulate the modest IL 6 elevations seen in most patients. (2) Conduct rigorous dose–response studies in human myotubes using physiological (pg/mL) rather than pharmacological (ng/mL) IL 6 concentrations. (3) Identify biomarkers (e.g., sIL 6R/sgp130 ratios, STAT3 phosphorylation status in muscle biopsies) that can stratify patients most likely to benefit from IL 6 targeted therapies. (4) Explore intermittent or exercise timed IL 6 blockade that spares the physiological surge after activity. (5) Combine IL 6 blockade with other modalities (e.g., myostatin inhibitors, exercise, nutrition) in multimodal trials.

## 7. Potential Therapeutic and Interventional Strategies

**Figure 7:**
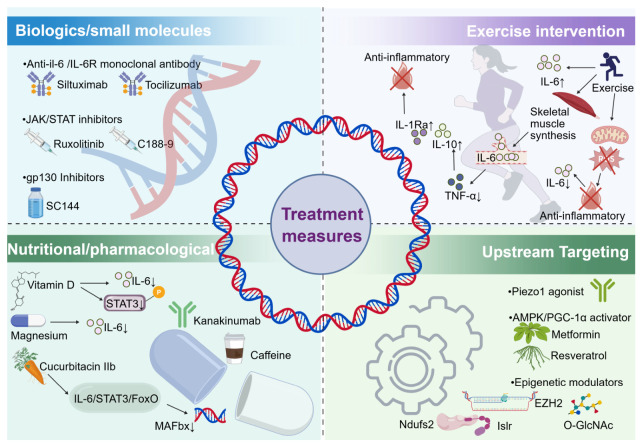
**Potential therapeutic interventions targeting the IL-6 signaling axis.** Strategies include: (1) biologics and small-molecule inhibitors directly blocking IL-6, IL-6R, or downstream JAK/STAT; (2) exercise prescriptions that harness physiological IL-6 surges and improve signaling homeostasis; (3) nutritional supplements and natural compounds that modulate IL-6 production or activity; (4) upstream regulators such as Piezo1 agonists, AMPK/PGC-1α activators, and epigenetic modifiers. An upward arrow represents an upward expression, and a downward arrow represents a downward expression.

The complex “dual-edged” role of interleukin-6 (IL-6) in skeletal muscle pathophysiology dictates that interventional strategies must be both precise and context-specific. The ideal therapeutic approach should not involve simply suppressing IL-6 globally. Instead, it should achieve fine-tuned regulation of its spatiotemporal signaling characteristics, its mode of action (classical signaling versus trans-signaling), and its downstream pathways. The goal is to preserve IL-6’s physiological functions—such as metabolic regulation and muscle regeneration—while curbing its pathological effects, including chronic inflammation and protein degradation. Thus, current interventional strategies are evolving toward multifaceted and multi-target development (Figure 7).

It is important to note that while preclinical studies have demonstrated efficacy of IL-6 pathway inhibitors in various animal models of muscle atrophy, the translatability of these findings depends critically on whether the animal models recapitulate human IL-6 dynamics. Many murine cachexia models (e.g., C26 tumor-bearing mice) produce supraphysiological IL-6 levels (1000+ pg/mL) that are rarely observed in human cancer patients [13]. Therefore, clinical trial design must account for the more modest IL-6 elevations typically seen in human disease.

### 7.1. Biologics and Small-Molecule Drugs Targeting the IL-6 Signaling Pathway

Directly targeting the IL-6 signaling axis represents the most specific approach in therapeutic intervention. This primarily involves drugs directed against the cytokine itself, its specific receptor, or key downstream kinases.

#### 7.1.1. Monoclonal Antibodies Targeting IL-6 or IL-6R

Monoclonal antibodies such as tocilizumab (anti-IL-6R) and siltuximab (anti-IL-6) are widely used in autoimmune diseases like rheumatoid arthritis. Their application provides a direct reference for treating skeletal muscle disorders. In preclinical models of skeletal muscle atrophy, these agents have demonstrated clear efficacy. For instance, in a denervation-induced muscle atrophy model, tocilizumab effectively inhibited the IL-6/JAK/STAT3 pathway and reduced the expression of autophagy-related genes. This alleviated muscle atrophy and the decline in mitochondrial mass [8]. Similarly, in an experimental autoimmune myasthenia gravis mouse model, IL-6R monoclonal antibody treatment, including tocilizumab, improved muscle weakness. It reduced IgG deposition at neuromuscular junctions and lowered serum levels of anti-acetylcholine receptor autoantibodies. This mechanism is associated with the suppression of follicular helper T cell and Th17 cell responses [28]. In cancer cachexia research, a pancreatic cancer model showed that tumor-derived IL-6 drives tissue wasting through a “dialogue” between adipose and muscle tissue. Depleting IL-6 significantly improved these symptoms [13]. However, extending these therapies to skeletal muscle diseases faces a key challenge: patient stratification. It is crucial to identify which patients (e.g., those with specific aberrantly activated IL-6 signaling subtypes or predominant trans-signaling) would benefit the most. This must be balanced against the risk of infections associated with systemic immunosuppression.

#### 7.1.2. JAK/STAT Inhibitors

As a core node downstream of IL-6 signaling, the JAK/STAT pathway offers another effective target for intervention. Ruxolitinib, a JAK1/2 inhibitor, has been shown in the C26 cancer cachexia model to alleviate muscle atrophy and improve grip strength. This effect is attributed to the inhibition of STAT3 phosphorylation and the expression of the downstream atrophy-related gene Atrogin-1 [30]. Similarly, in denervation models, the STAT3-specific inhibitor C188-9 effectively rescues muscle atrophy to a degree comparable to that of IL-6R antibodies [8]. Such small-molecule drugs offer the advantage of convenient oral administration and can simultaneously block the effects of multiple cytokines that utilize the JAK/STAT pathway, such as IL-11 and leukemia inhibitory factor. However, their lack of specificity may also lead to side effects, including anemia and thrombocytopenia. Therefore, developing muscle-targeted JAK/STAT inhibitors or exploring more selective STAT3 dimerization inhibitors (e.g., C188-9) represents an important direction for improving the therapeutic window.

#### 7.1.3. gp130 Inhibitors

Inhibitors targeting the shared signal-transducing receptor gp130, such as SC144, theoretically block both the classical and trans-signaling of IL-6. This approach may exhibit greater efficacy against pathological conditions dominated by trans-signaling, including certain chronic inflammatory diseases and cancers. However, gp130 serves as a common receptor for multiple IL-6 family cytokines, such as cardiotrophin-1 and oncostatin M. Its broad inhibition could interfere with essential physiological processes, including cardioprotection and neurodevelopment, leading to unforeseen systemic side effects [57]. Therefore, this strategy currently serves primarily as a research tool. Its clinical translation would require highly selective tissue targeting or localized delivery systems.

### 7.2. Exercise Intervention

Exercise serves as a “natural medicine” for regulating IL-6 levels. Its role extends beyond mere anti-inflammatory effects, embodying a physiological reshaping of IL-6 spatiotemporal dynamics. This positions exercise as central to maintaining metabolic and immune balance. Acute exercise induces a beneficial IL-6 peak: Moderate- to high-intensity exercise, particularly exhaustive or prolonged endurance exercise, triggers significant synthesis and release of IL-6 from skeletal muscle into the circulation. This process typically occurs without a concomitant rise in classic inflammatory cytokines like TNF-α [62]. This exercise-induced IL-6 surge holds multiple physiological significances. It acts as an energy sensor, promoting hepatic gluconeogenesis and adipose tissue lipolysis to maintain blood glucose stability. It also enhances insulin sensitivity and glucose uptake by activating AMPK and PI3K-Akt pathways in skeletal muscle and liver [5]. Furthermore, elevated myogenic IL-6 can inhibit the production of pro-inflammatory factors like TNF-α and induce the release of anti-inflammatory cytokines such as IL-1Ra and IL-10. Consequently, it creates a transient anti-inflammatory internal milieu [132]. Therefore, acute exercise-induced IL-6 release is a crucial physiological signal for the body to adapt to metabolic demands and maintain homeostasis.

Long-term exercise training improves IL-6 signaling homeostasis: Regular exercise fundamentally optimizes the body’s response to IL-6. On one hand, training reduces the basal level of chronic low-grade inflammation by increasing muscle mass, improving mitochondrial function (e.g., upregulating PGC-1α expression), and enhancing antioxidant capacity. This leads to decreased systemic IL-6 concentration at rest [14]. On the other hand, exercise training increases tissue sensitivity to the metabolic benefits of IL-6. Studies show that training can reverse the inhibition of mTORC1 signaling and mitochondrial dysfunction caused by IL-6 overexpression, thereby helping restore muscle anabolic capacity [50,58]. Thus, long-term exercise reshapes the physiological role of IL-6 through these dual mechanisms, establishing its interventional value in metabolic diseases.

Personalization of exercise prescription is key to precise intervention: Different exercise modalities differentially impact IL-6, providing a basis for individualized strategies. Resistance training primarily improves inflammatory status long-term by promoting muscle hypertrophy and increasing basal metabolic rate. In contrast, high-intensity interval training often elicits a more pronounced acute IL-6 response. The associated inflammation and oxidative stress are closely related to the design of work-rest intervals [133]. For individuals with existing muscle atrophy or chronic diseases, exercise intensity and duration require careful regulation. This is to avoid excessive muscle damage and paradoxical strong inflammatory responses from overtraining. Therefore, an “exercise prescription” should be systematic and individualized, much like a drug prescription. It must tailor the exercise mode, intensity, frequency, and duration based on an individual’s physiological status, disease stage, and training goals.

Future research needs to further decipher the molecular networks of IL-6 in different exercise contexts. It should also establish biomarker-based personalized exercise prescription systems. This will better harness the regulatory potential of exercise and IL-6 for muscle health and disease intervention.

### 7.3. Nutritional and Pharmacological Interventions

Modulating IL-6-related metabolic and inflammatory pathways through dietary supplementation or specific compounds represents a feasible auxiliary strategy.

#### 7.3.1. Nutrient Supplementation

The vitamin D receptor is widely expressed in skeletal muscle. Exercise can upregulate its expression, subsequently inhibiting IL-6 mRNA expression and STAT3 phosphorylation, thereby potentially buffering against potential excessive post-exercise inflammation [134]. Clinical observations further indicate that vitamin D deficiency is associated with muscle weakness and a high-inflammatory state. Vitamin D supplementation not only helps improve muscle function but also modulates IL-6 levels. Therefore, vitamin D is not only a key nutrient for maintaining muscle function, but its receptor activation can also synergize with exercise to jointly suppress IL-6 expression and STAT3 phosphorylation [135,136]. This demonstrates its dual role in regulating the inflammatory microenvironment of skeletal muscle. Furthermore, magnesium serves as a cofactor for numerous enzymes and is involved in energy metabolism and inflammation regulation. A study on runners showed that one-week magnesium supplementation attenuated the post-exercise increase in serum IL-6 following downhill running. It also accelerated the relief of muscle soreness and the recovery of blood glucose levels [137]. This suggests that magnesium may promote post-exercise functional recovery by modulating inflammatory responses and glucose metabolism, providing a rationale for its intervention in exercise-related muscle damage. On the other hand, metabolites of ω-3 polyunsaturated fatty acids, such as protectin D1, have been found to act synergistically with IL-6. This combination improves insulin sensitivity in obese diabetic model mice [138]. Collectively, these studies reveal the feasibility and potential value of regulating IL-6 signaling through nutritional interventions in the pathophysiological processes of skeletal muscle. They lay a foundation for developing multi-target, comprehensive nutritional support strategies.

#### 7.3.2. Natural Products and Drug Repurposing

Given the significant role of IL-6 in the pathophysiology of skeletal muscle, multiple targeted intervention strategies have been developed. For example, the natural compound cucurbitacin IIb can modulate the IL-6/STAT3/FoxO signaling pathway and down-regulate the expression of the muscle atrophy gene MAFbx, thereby alleviating muscle wasting in a cancer cachexia model [32]. This provides experimental evidence for using natural products to regulate pathways related to muscle atrophy. In addition, the anti-IL-1β monoclonal antibody canakinumab effectively suppresses IL-1β-induced IL-6 secretion in myotubes derived from patients with Duchenne muscular dystrophy [128]. These findings not only offer new insights into treating inflammatory myopathies driven by the IL-1β/IL-6 axis but also suggest a lower risk and higher translational potential for repurposing FDA-approved drugs in muscle disorders. Meanwhile, daily dietary components such as caffeine have been found to stimulate skeletal muscle to produce IL-6, which subsequently activates hepatic STAT3 signaling and ameliorates non-alcoholic fatty liver disease [46]. This discovery highlights the critical role of myogenic IL-6 in mediating muscle–liver metabolic crosstalk, implying that certain food constituents may exert systemic metabolic benefits by modulating local IL-6 levels.

A common advantage of these interventions lies in their pleiotropic effects, often simultaneously influencing multiple pathophysiological processes closely intertwined with IL-6 signaling, such as oxidative stress, mitochondrial function, and even gut microbiota. Therefore, future research urgently requires more high-quality clinical trials to systematically verify the precise efficacy, safety, and optimal dosage of specific nutritional formulations or natural compounds in preventing and treating sarcopenia, cachexia, and related conditions.

### 7.4. Targeting Upstream Regulatory Mechanisms

Targeting key upstream molecules that regulate IL-6 production or determine its pathological effects may enable more precise and root-cause therapeutic interventions.

#### 7.4.1. Targeting Mechano-Chemical Signal Transduction: The Piezo1 Channel

Studies have shown that the decline in basal cytosolic calcium concentration in skeletal muscle during disuse atrophy is associated with the downregulation of the mechanosensitive channel Piezo1. Acute disruption of Piezo1 upregulates KLF15 and IL-6, inducing muscle atrophy. However, antibody-mediated blockade of IL-6 can prevent this atrophy [26]. This reveals a novel pathway: “reduced mechanical stimulation → Piezo1 downregulation → decreased basal calcium signaling → activation of the KLF15/IL-6 axis → muscle atrophy.” Therefore, developing Piezo1 channel agonists or strategies to maintain basal calcium signaling in disused muscle represents a highly promising new direction for preventing disuse atrophy.

#### 7.4.2. Targeting Energy and Mitochondrial Sensors: AMPK and PGC-1α

AMPK is a primary sensor of cellular energy status, while PGC-1α is a master regulator of mitochondrial biogenesis and function. Activating the AMPK/PGC-1α axis can improve mitochondrial function and reduce mitochondrial reactive oxygen species (ROS) production. This, in turn, indirectly suppresses the activation of pro-inflammatory pathways such as NF-κB and the production of pathological IL-6. In sepsis models, IL-6 deficiency alleviates muscle atrophy by upregulating PGC-1α and inhibiting mitochondrial ROS production [14]. Consequently, using AMPK activators like metformin, or employing exercise training/specific compounds (e.g., resveratrol) to activate this axis, offers a broad-spectrum metabolic strategy to counteract IL-6-mediated muscle wasting.

#### 7.4.3. Targeting Epigenetic and Transcriptional Regulation

Recent studies have unveiled the complex upstream regulatory networks controlling IL-6 expression. For instance, in aged muscle stem cells, a reduction in the histone H3K27me3 repressive mark leads to increased NF-κB1 expression. This, in turn, drives the secretion of IL-6 and SPP1, promoting the proliferation of fibro-adipogenic progenitors and muscle fibrosis [125]. This suggests that targeting specific epigenetic modifiers (such as EZH2) or transcription factors could correct aberrant IL-6 expression programs at their source. Additionally, O-GlcNAcylation in muscle cells can upregulate IL-6 expression by enhancing p65 activity and nuclear translocation in response to cold stress [27]. This represents another potential regulatory node involving nutrient sensing.

#### 7.4.4. Targeting Mediators of Muscle-Organ Crosstalk

IL-6 acts as a key endocrine messenger in the dialogue between muscle and other tissues, including adipose tissue, liver, bone, and even the brain. For example, muscle-derived IL-6 can influence thermogenesis and energy expenditure in brown adipose tissue. It does this by modulating the interaction between the Ig superfamily protein Islr and the mitochondrial complex I subunit Ndufs2 [47]. Intervening in these tissue-specific communication mediators, rather than systemic IL-6 signaling, may enable more precise metabolic modulation. This approach could also avoid the side effects associated with systemic inhibition.

In summary, interventions targeting IL-6 in skeletal muscle diseases have evolved into a multifaceted strategic framework. This network spans from direct targeting and lifestyle modifications to upstream mechanistic interventions. Future challenges lie in deepening our understanding of the spatiotemporal, cellular source, and pathological context specificities of IL-6 signaling. Building on this knowledge, developing precise biomarkers for patient stratification will be crucial for achieving personalized therapy. Furthermore, determining how to optimally combine pharmacological interventions with non-pharmacological approaches like exercise and nutrition to create synergistic regimens will be key to improving long-term patient outcomes.

### 7.5. Synthesis and Commentary

Section 7 presents a comprehensive therapeutic landscape targeting the IL 6 axis, ranging from biologics (tocilizumab, siltuximab) and small molecules (ruxolitinib, C188 9) to non-pharmacological interventions (exercise, nutrition) and upstream modulators (Piezo1 agonists, AMPK activators). The clinical validation status of each approach varies considerably (summarized in Table 1). Tocilizumab and ruxolitinib are FDA-approved for other indications, but neither is approved for muscle wasting; their use in muscle atrophy remains off-label and experimental. Olamkicept (sgp130Fc) selectively blocks trans signaling and has shown efficacy in inflammatory bowel disease, but has not been tested in muscle conditions [112]. Exercise and nutritional strategies are clinically implemented as standard supportive care, but their effects on IL 6 signaling are indirect and pleiotropic.

Critical questions for translation include: (1) Should we aim for complete IL 6 neutralization or selective trans signaling blockade? The latter is theoretically safer because it preserves classical signaling (e.g., exercise-induced metabolic benefits, host defense). sgp130Fc is promising but requires human trials in muscle wasting. (2) What is the optimal timing and duration of therapy? Chronic blockade may increase infection risk (e.g., upper respiratory tract infections with tocilizumab). Intermittent or peri-exercise dosing might be considered. (3) Can we repurpose FDA-approved JAK inhibitors (ruxolitinib, baricitinib, tofacitinib) for muscle atrophy? Ruxolitinib has shown efficacy in denervation and cancer cachexia models [8,30], but systemic JAK inhibition has significant hematological and immunological side effects that may limit long-term use. Muscle-targeted delivery (e.g., via nanoparticles or conjugation with muscle-homing peptides) could improve the therapeutic window. (4) Should we target upstream regulators (Piezo1, AMPK) rather than IL 6 itself? This might restore homeostasis more physiologically, but such approaches are still at an early preclinical stage. (5) How do we design clinical trials that account for patient heterogeneity? Biomarker-stratified trials (e.g., selecting patients with high sIL 6R or pSTAT3 in muscle) are essential to demonstrate efficacy in responsive subgroups.

## 8. Conclusions and Future Perspectives

In summary, IL-6 plays a complex and central role in skeletal muscle biology. Its functional spectrum ranges from a “guardian” that maintains metabolic homeostasis and promotes regeneration to a “destroyer” that drives cachectic diseases. This seemingly paradoxical dual identity is not random. Instead, it is determined by a series of finely regulated factors. These primarily include the spatiotemporal specificity of signaling pathways, the cellular and molecular composition of the acting microenvironment, and the overall physiological or pathological state of the organism. For instance, IL-6 induced by acute exercise and produced via muscle autocrine/paracrine mechanisms exerts positive metabolic regulation and pro-regenerative effects. It enhances glucose uptake, insulin sensitivity, and protein synthesis by activating pathways such as AMPK/PI3K-Akt-mTOR. In contrast, under chronic disease conditions—such as cancer, sepsis, or chronic kidney disease—persistently elevated IL-6, often derived from immune cells or other tissues and particularly signaling via the trans-signaling pathway, leads to detrimental outcomes. It excessively activates pathways like JAK/STAT3 within skeletal muscle. This results in proteasome system activation, dysregulated autophagy, and mitochondrial dysfunction, ultimately causing severe muscle atrophy. Therefore, a deeper understanding of the dual roles of IL-6 not only significantly enriches our fundamental knowledge of muscle physiology and pathology. It also opens a promising avenue for developing novel therapeutic strategies against a range of debilitating muscle disorders.

However, translating this knowledge into effective clinical interventions remains a significant challenge. Future research must achieve breakthroughs across several frontiers.

Firstly, elucidating the precise “mechanistic switch” that controls the functional transition of IL-6 is a core future challenge. Research must move beyond simply measuring IL-6 levels and instead deeply dissect the molecular basis of its functional shift within specific pathological contexts. For example, it is crucial to identify which upstream signals (such as Piezo1-mediated downregulation of calcium signaling) and microenvironmental factors (like specific cytokine combinations or oxidative stress levels) determine the switch of IL-6 signaling from physiological to pathological during muscle atrophy. Research should aim to identify key molecular “switching points.” These could include specific post-translational modifications, changes in receptor complex composition (such as the balance between membrane-bound IL-6R and soluble IL-6R), or a shift in downstream signaling nodes (like the balance between STAT3 and STAT1 activation). Recent studies indicate that sustained activation of the IL-6/JAK/STAT3 pathway is a key driver of atrophy in denervation or cancer cachexia models, and inhibiting this pathway effectively mitigates muscle wasting. Therefore, clarifying under which microenvironmental contexts the persistent activation of STAT3 overwhelms other beneficial signals is a vital direction for identifying this “switch.”

Secondly, developing precise targeting strategies based on mechanistic understanding is paramount. Current clinical IL-6 pathway inhibitors (such as tocilizumab and siltuximab), while effective for certain systemic inflammatory diseases, may disrupt IL-6’s physiological functions due to systemic blockade. This includes potentially affecting post-exercise metabolic benefits or tissue repair. Consequently, future drug development should focus on distinguishing between classical and trans-signaling of IL-6. Since trans-signaling is believed to dominate in pathological processes, designing drugs that selectively inhibit the formation or function of the sIL-6R/gp130 complex (e.g., olamkicept) might allow safer treatment of muscle-wasting diseases without impairing classical signaling-mediated host defense and regenerative functions. Additionally, developing prodrugs or conditional antibodies activated only in specific pathological environments (such as low pH or high ROS conditions) is a promising strategy for achieving precise targeting and reducing off-target effects.

Thirdly, integrating multi-omics and systems biology approaches will provide an unprecedented panoramic view. Future efforts should leverage high-throughput technologies like single-cell RNA sequencing, spatial transcriptomics, proteomics, and metabolomics. These tools should be used to comprehensively map the IL-6 signaling network and its dynamic interactions with other key pathways (such as TGF-β, Myostatin/GDF-11, IGF-1/Akt) across different disease models and patient populations. This systems-level analysis will aid in discovering novel, more specific biomarkers for early diagnosis and treatment efficacy monitoring. More importantly, it can reveal entirely new drug targets. Examples include IL-6-regulated non-coding RNAs (e.g., miR-497-5p), epigenetic regulators, or specific metabolic enzymes, all of which could become new avenues for intervening in muscle pathology. Recent studies using such methods have already uncovered novel regulatory networks in models like cancer cachexia.

Fourthly, exploring and optimizing combination therapies will likely be key to overcoming complex muscle diseases. Given the multifactorial nature of muscle atrophy, single-target therapies often have limited efficacy. Combining IL-6-targeting drugs with other interventions may yield synergistic or additive effects. For instance, combining an IL-6 pathway inhibitor with an antibody targeting myostatin (e.g., Domagrozumab) could simultaneously inhibit catabolism and promote anabolism. Furthermore, non-pharmacological interventions are crucial. Planned resistance exercise itself is a powerful stimulus for inducing beneficial myokine secretion and countering atrophy. Personalized nutritional support (e.g., supplementing with branched-chain amino acids, vitamin D, or omega-3 fatty acids) can provide substrates for muscle repair and modulate the inflammatory milieu. Therefore, future clinical trial designs should more frequently consider such multimodal combination strategies. They should also utilize biomarkers for patient stratification to achieve personalized treatment.

The dual role of IL 6 in skeletal muscle–as a beneficial myokine (exercise, regeneration, metabolic adaptation) and as a pathological driver (cachexia, sarcopenia, denervation atrophy)–is now firmly established. However, the field is at a critical juncture, moving from phenomenological description to mechanistic precision. The overarching future challenge is to understand how the four key parameters–context, concentration, duration, and signaling topology–integrate to determine IL 6’s final biological output in a given pathophysiological setting.

Based on the synthesis of the entire review, we propose the following research priorities:

1. Single cell and spatial omics of human skeletal muscle across disease states (cancer cachexia, sarcopenia, denervation, DMD) to map IL 6 producing cells and IL 6 responsive cells at unprecedented resolution. This will resolve the muscle-derived vs. immune-derived controversy.
2. Development of human muscle organoid and microphysiological systems that recapitulate the IL 6 signaling environment (including classical and trans signaling, and co-culture with immune cells) and allow screening of therapeutics under clinically relevant IL 6 concentrations.
3. Conditional, cell-type-specific IL 6 and gp130 knockout models in mice to disentangle autocrine, paracrine, and endocrine effects. Muscle-specific IL 6 knockout has been informative [7], but equivalent tools for immune cells, FAPs, and satellite cells are needed.
4. Biomarker-driven clinical trials of IL 6 pathway inhibitors (tocilizumab, ruxolitinib, olamkicept) in muscle wasting, with pre-specified subgroup analyses based on muscle pSTAT3, serum sIL 6R, or genetic polymorphisms (e.g., rs1800795). The first such trial in cancer cachexia is underway (NCT05349149) [130], but results are pending.
5. Combination therapies that simultaneously target IL 6 and other atrophy pathways (myostatin, activin, TNF-α, glucocorticoid receptors) or combine pharmacological blockade with exercise/nutritional support to preserve anabolic signaling.
6. Exploration of “biased signaling”–developing ligands that preferentially activate the PI3K Akt metabolic pathway over the JAK/STAT3 atrophic pathway. This remains a distant but potentially transformative goal.

In conclusion, IL 6 is neither a simple “friend” nor “foe”. It is a context-dependent master regulator whose roles are dictated by the integrated sum of concentration, duration, source, and signaling mode. Achieving “precision modulation” of the IL 6 system–harnessing its beneficial actions while suppressing its pathological effects–will require a deep mechanistic understanding and innovative therapeutic designs. The next decade will determine whether this ancient cytokine can be tamed to serve human muscle health across the lifespan.

## Figures and Tables

**Table 1:** Therapeutic Strategies Targeting IL-6 Signaling in Skeletal Muscle: Clinical Validation Status.

Strategy	Examples	Clinical Status	Evidence in Skeletal Muscle	Key References
**Clinically Approved (Non-muscle indications)**				
Anti-IL-6R mAb	Tocilizumab	FDA-approved for rheumatoid arthritis, juvenile idiopathic arthritis	Preclinical efficacy in denervation atrophy, myasthenia gravis models	[8,28]
Anti-IL-6 mAb	Siltuximab	FDA-approved for multicentric Castleman’s disease	Preclinical in cancer cachexia models	[13]
JAK1/2 inhibitor	Ruxolitinib	FDA-approved for myelofibrosis, polycythemia vera	Preclinical efficacy in denervation and cancer cachexia	[8,30]
**In Clinical Trials for Muscle-Related Conditions**				
Anti-IL-1β mAb	Canakinumab	Phase II/III for various inflammatory diseases	Ex vivo efficacy in DMD patient myotubes	[128]
gp130-targeting	Olamkicept (sgp130Fc)	Phase II for inflammatory bowel disease	Preclinical in myopia-related muscle remodeling	[112]
**Experimental (Preclinical Only)**				
STAT3 inhibitors	C188-9	Preclinical	Efficacy in denervation atrophy	[8]
Piezo1 modulators	Yoda1 (agonist)	Preclinical	Potential for disuse atrophy prevention	[26]
Natural products	Cucurbitacin IIb	Preclinical	Efficacy in cancer cachexia models	[32]
AMPK activators	Metformin, AICAR	Approved for diabetes (metformin)	Preclinical evidence for muscle protection	[14]
**Non-Pharmacological (Clinically Implemented)**				
Exercise training	Aerobic, resistance	Standard of care	Clinical evidence for metabolic benefits	[2,108]
Nutritional	Vitamin D, magnesium, leucine	Standard supportive care	Clinical evidence for inflammation reduction	[4,137]

Abbreviations: DMD, Duchenne muscular dystrophy; FDA, U.S. Food and Drug Administration; mAb, monoclonal antibody.

## Data Availability

No new data were created or analyzed in this study.
